# Feruloyl Esterases for Biorefineries: Subfamily Classified Specificity for Natural Substrates

**DOI:** 10.3389/fbioe.2020.00332

**Published:** 2020-04-23

**Authors:** Emilie N. Underlin, Matthias Frommhagen, Adiphol Dilokpimol, Gijs van Erven, Ronald P. de Vries, Mirjam A. Kabel

**Affiliations:** ^1^Laboratory of Food Chemistry, Wageningen University & Research, Wageningen, Netherlands; ^2^Department of Chemistry, Technical University of Denmark, Lyngby, Denmark; ^3^Fungal Physiology, Westerdijk Fungal Biodiversity Institute and Fungal Molecular Physiology, Utrecht University, Utrecht, Netherlands

**Keywords:** biomass, corn stover, feruloyl esterases, hydroxycinnamic acids, lignin-carbohydrate complex, pectin, wheat straw, xylan

## Abstract

Feruloyl esterases (FAEs) have an important role in the enzymatic conversion of lignocellulosic biomass by decoupling plant cell wall polysaccharides and lignin. Moreover, FAEs release anti-oxidative hydroxycinnamic acids (HCAs) from biomass. As a plethora of FAE candidates were found in fungal genomes, FAE classification related to substrate specificity is an indispensability for selection of most suitable candidates. Hence, linking distinct substrate specificities to a FAE classification, such as the recently classified FAE subfamilies (SF), is a promising approach to improve the application of these enzymes for a variety of industrial applications. In total, 14 FAEs that are classified members of SF1, 5, 6, 7, 9, and 13 were tested in this research. All FAEs were investigated for their activity toward a variety of substrates: synthetic model substrates, plant cell wall-derived substrates, including lignin, and natural substrates. Released HCAs were determined using reverse phase-ultra high performance liquid chromatography coupled to UV detection and mass spectrometry. Based on this study, FAEs of SF5 and SF7 showed the highest release of FA, *p*CA, and diFAs over the range of substrates, while FAEs of SF6 were comparable but less pronounced for diFAs release. These results suggest that SF5 and SF7 FAEs are promising enzymes for biorefinery applications, like the production of biofuels, where a complete degradation of the plant cell wall is desired. In contrast, SF6 FAEs might be of interest for industrial applications that require a high release of only FA and *p*CA, which are needed as precursors for the production of biochemicals. In contrast, FAEs of SF1, 9 and 13 showed an overall low release of HCAs from plant cell wall-derived and natural substrates. The obtained results substantiate the previous SF classification as a useful tool to predict the substrate specificity of FAEs, which eases the selection of FAE candidates for industrial applications.

## Introduction

Plant polysaccharides within lignocellulosic biomass are considered a sustainable and green resource for the production of biobased chemicals and fuels after depolymerization (Mood et al., 2013; Loqué et al., 2015). The major component of lignocellulosic biomass consists of the plant cell wall (PCW) – a network of cellulose, hemicellulose and lignin. This complex structure hinders the depolymerization of the PCW by, for example, fungal enzymes (Kabel et al., 2007; Pauly and Keegstra, 2008). Hence, enzymes that are able to increase the PCW accessibility, such as feruloyl esterases (FAEs), are widely applied in biorefinery processes for the biofuel, food, feed, pulp, and paper industries (Haruhiko et al., 2007; Shuichi et al., 2009; West et al., 2009; Hiroyuki et al., 2010; Nsereko et al., 2010a,b,c; West and William, 2010).

The PCW consists of a primary and a secondary layer, which have different compositions. The primary layer is mainly composed of cellulose, hemicelluloses such as xyloglucan, and various types of pectins (Carpita and Gibeaut, 1993; Hoffman et al., 2005; Carpita and McCann, 2008). The secondary layer is mainly composed of cellulose, which is embedded in a network consisting of hemicellulosic xylan and/or mannan, and the aromatic polymer lignin (Carpita and McCann, 2008; Harris and Stone, 2008). Beyond the complexity of the PCW polymers, the accessibility of enzymes is hindered by various cross-links, linking either two polysaccharide chains with each other or a polysaccharide chain to lignin. One of the major cross-links in the PCW is formed via one or two hydroxycinnamic acid (HCA) units, in particular ferulic acid (FA) (Harris and Stone, 2008).

**FIGURE 1:**
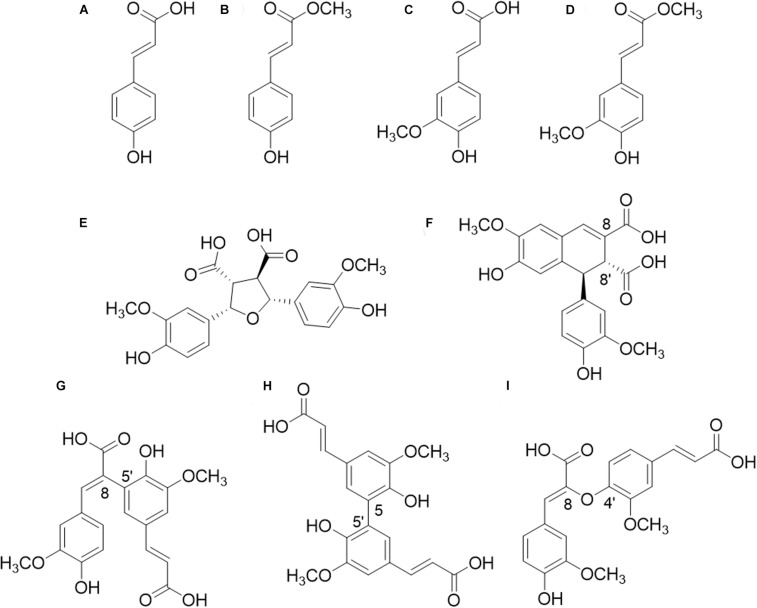
Structures of hydroxycinnamic acids (HCAs). **(A)** p-coumaric acid (pCA); **(B)** methyl p-coumarate; **(C)** ferulic acid (FA); **(D)** methyl ferulate; **(E)** 8-8′-(furan)-diferulic acid (8-8′-diFA); **(F)** 8-8′-(aryl)-diFA; **(G)** 8-5′-diFA; **(H)** 5-5′-diFA; **(I)** 8-O-4′-diFA.

In xylan-rich PCW, such as in *Gramineae* (i.e., corn fiber, various grasses), the carboxyl group of FA (Figure 1) is ester-linked to the *O*-5 position of α-L-arabinofuranosyl substituents of the xylan backbone (de O Buanafina, 2009; Harris and Trethewey, 2010). In pectin-rich PCWs on the other hand, e.g., the roots of various dicots (i.e., sugar beet), the carboxyl group of FA is ester linked to the *O*-6 position of β-D-galactopyranosyl residues in galactan and/or to the *O*-2 and *O*-5 positions of α-L-arabinofuranosyl residues in arabinan (Ralet et al., 1994; Levigne et al., 2004a, b). Furthermore, various diferulic acids (diFAs) and triferulic acids (triFAs) are esterified to PCW polysaccharides, of which 8-8′-(tetrahydrofuran)-diFA (8-8′-(furan)-diFA), 8-5′-diFA, 5-5′-diFA, and 8-*O*-4′-diFA are the most common in the PCW (Figure 1; Dilokpimol et al., 2016). A part of these mono-, di-, and tri-ferulic acid compounds can be linked to another polysaccharide chain or is etherified (or esterified) to lignin, which leads to the formation of cross-links (Ralph et al., 1994; de O Buanafina, 2009; Vismeh et al., 2013; de Oliveira et al., 2015; Dilokpimol et al., 2016; Hatfield et al., 2017). Besides FA, another esterified HCA is *p*-coumaric acid (Figure 1), which is mostly reported to be *γ*-esterified to *β-O*-4-linkages within the lignin macromolecule, in particular in the PCW of certain *Gramineae* (Ralph, 2010). Small amounts of FA could also be ester-linked to lignin for which further evidence has yet to be obtained (Regner et al., 2018).

FAEs (EC 3.1.1.73) represent a subclass of the carbohydrate esterase family 1 (CE1) from the CAZy database (CAZy.org), which release HCAs from plant biomass via the hydrolysis of the ester-linkages (Wong, 2006; Topakas et al., 2007; Faulds, 2010; Lombard et al., 2014). This hydrolysis of cross-links between polysaccharides and lignin increases the accessibility of PCW polysaccharides for other enzymes, such as glycosyl hydrolases (GH). The released HCAs are valuable precursors for the production of biochemicals, like antioxidants, that are applied in the cosmetic and pharmaceutical industry (de O Buanafina, 2009; de Oliveira et al., 2015; Dilokpimol et al., 2016).

Genomes among the fungal kingdom comprise a plethora of FAE candidates. As a result, a classification of FAEs, which is preferably coupled to their substrate specificity, is indispensable for the selection of suitable FAE candidates for industrial applications. Several classifications of FAEs have been suggested (Crepin et al., 2004; Benoit et al., 2008; Udatha et al., 2011). The most commonly used is the ABCD classification, which was introduced by Crepin et al. (2004). This classification was based on the substrate specificity of a limited number of FAEs toward the four common methylated synthetic model substrates (methyl ferulate, methyl *p*-coumarate, methyl caffeate, and methyl sinapate) and the ability of FAEs to release diFAs from isolated PCW hemicelluloses (Crepin et al., 2004). Within the past decade, it has become apparent that more and more characterized FAEs could not be categorized in this system, which indicates that the ABCD classification is limited in its ability to reflect the broad substrate specificity and diversity of the FAEs (Dilokpimol et al., 2016). Hence, a new amino acid sequence-based classification system of these enzymes has been designed, which divides (putative) FAEs into 13 subfamilies (SF) and expands the more limited SF classification that previously described seven SFs (Benoit et al., 2008; Dilokpimol et al., 2016).

This SF classification is based on phylogenetic relationships and sequence homologies of over 1000 putative fungal FAEs. Briefly, these FAE amino acid sequences were collected by BLASTP search against over 200 fungal genomes published before 2014 using 20 amino acid sequences (Dilokpimol et al., 2016). The resulting sequences were aligned using Multiple Sequence Comparison by Log-Expectation (MUSCLE). The phylogenetic relationship was analyzed by using the neighbor-joining method. The phylogenetic relationship also showed that FAEs evolved from highly divert esterase families including tannases (SF1-4), acetyl xylan esterases (SF6), lipases (SF7), and choline esterases (SF12-13) (Dilokpimol et al., 2016). In a later study, 20 representative FAEs covering 11 SFs were biochemically characterized using four common synthetic model substrates (Dilokpimol et al., 2018). However, it has not been assessed how FAEs of different SFs hydrolyze ester-linked HCAs from natural plant substrates. Therefore, in this study, we investigated the natural plant substrate specificity of 14 fungal FAEs from different SFs (SF1, 5, 6, 7, 9, and 13) (Table 1). Until today, only the five SF5 and three SF6 members have been classified in the CAZY database – as carbohydrate esterase family 1 members (Lombard et al., 2014). PCW-derived and natural substrates from different species were used to mimic the ability of these FAEs to release HCAs (including monomers, dimers and trimers) during biomass degradation in nature and to emphasize the potential of the 14 FAEs for diverse industrial applications. In detail, the PCW-derived substrates sugar beet pectin (SBP), corn fiber oligosaccharides (CFoligo), insoluble arabinoxylans (WAX-i), lignin isolate from corn stover (CSlignin), and the natural substrates corn stover (CS) and wheat straw (WS) were tested.

**TABLE 1:** Overview of FAEs employed in this study including fungal origin, accession number, name identification, classification according to the SF and ABCD system.

Fungal species	Accession number	Name	SF classification^†,‡^	ABCD-classification	pH Optimum	CAZy classification^§^	References
*Aspergillus niger*	CAC83933.1	AnFaeB	1	B	5	Not in CAZy	de Vries et al., 2002
*Aspergillus sydowii*	Aspsy1_293049	AsFaeF	1	B	6	Not in CAZy	Dilokpimol et al., 2018
*Aspergillus niger*	XP_001395336.1	AnFaeC	5	C	6	CE1	Dilokpimol et al., 2017
*Aspergillus nidulans*	AN5267	AnidFAEC	5	C or D	5	CE1	Debeire et al., 2012
*Aspergillus sydowii*	Aspsy1_154482	AsFaeC	5	C or D	6	CE1	Dilokpimol et al., 2018
*Chrysosporium lucknowense* C1	JF826027	C1FaeA1	5	A	7	CE1	Kühnel et al., 2012
*Chrysosporium lucknowense* C1	JF826028	C1FaeA2	5	A	7.5	CE1	Kühnel et al., 2012
*Fusarium oxysporum*	Fusox1_8990	FoFae2	6	n/a	6	CE1	Dilokpimol et al., 2016
*Aspergillus sydowii*	Aspsy1_1158585	AsFaeE	6	C or D	6	CE1	Dilokpimol et al., 2018
*Chrysosporium lucknowense* C1	JF826029	C1FaeB2	6	B	7	CE1	Kühnel et al., 2012
*Aspergillus niger*	CAA70510.1	AnFaeA	7	A	5	Not in CAZy	de Vries et al., 1997
*Aspergillus niger*	An15g05280	AnFaeJ	9	n/a	5	Not in CAZy	Dilokpimol et al., 2016
*Aspergillus sydowii*	Aspsy1_160668	AsFaeI	13	B	6	Not in CAZy	Dilokpimol et al., 2018
*Stereum hirsutum*	Stehi1_73641	ShFae1	13	n/a	n/a	Not in CAZy	Dilokpimol et al., 2016

*^†^SF classification as according to the phylogenetic tree made in 2016 (Dilokpimol et al., 2016). ^‡^Based on reactivity toward the four most common methyl esters according to Crepin et al. (2004). ^§^Based on CAZy classification according to Lombard et al. (2014). n/a, not applicable.*

Released HCAs were identified and determined by using RP-UHPLC-UV-ESI-MS/MS. The obtained results substantiate that the SF classification is a useful tool to predict the substrate specificity of FAEs, which eases the selection of FAE candidates for industrial applications.

## Materials and Methods

### Substrates

Methyl ferulate, methyl *p*-coumarate, ferulic acid, and *p*-coumaric acid were obtained from Apin Chemicals Ltd. (Abingdon, United Kingdom), Carbosynth (Compton, United Kingdom), Fluka^TM^ (Leicestershire, United Kingdom) and Sigma Aldrich (Darmstadt, Germany), respectively. Sugar beet pectin (SBP, Pectin Betapec RU301) was purchased from Herbstreith & Fox KG (Neuenbürg, Germany). Wheat arabinoxylan insoluble was obtained from (WAX-i, Megazyme, Bray, Ireland) and the corn fiber oligomers (CFoligo)-preparation was obtained as previously described (Appeldoorn et al., 2010). Corn stover (CS) was obtained from DSM (Delft, The Netherlands) and wheat straw (WS) were kindly provided by CNC Grondstoffen B.V. (Milsbeek, The Netherlands). Prior to incubation, both CS and WS were ball milled as previously described (van Erven et al., 2017), giving rise to deconstruction of cellulose crystallinity and making the substrates more susceptible to enzymatic pretreatment (Broxterman et al., 2018). The carbohydrate content and composition of all substrates used was performed as previously described (Englyst and Cummings, 1984). The analysis details are presented in the supporting information (Supplementary Table S1).

### Washing of CS and WS

Around 10 mg of ball mill CS and WS was washed with 1 mL water in a head-over-tail shaker for 15 min at 20°C. Afterward, all samples were centrifuged at (12,000 *g*, 15 min, 10°C). The supernatant was removed and the obtained pellet was washed another two times under the same conditions. The washed pellet was freeze dried and the weight loss (water soluble CS and WS) was calculated. Experiments were performed in triplicate. Results are given in the Discussion section.

### Preparation and Characterization of the Corn Stover Lignin Isolate

Corn stover lignin isolate (CSlignin) was obtained as previously described for wheat straw lignin and analyzed for residual carbohydrate content. Briefly, lignin was isolated from extractive-free planetary ball-milled corn stover by aqueous dioxane, freeze-dried and subsequently enzymatically purified (van Erven et al., 2017). Structural analysis of the CSlignin was performed by using 2D heteronuclear single quantum coherence (HSQC) NMR; (van Erven et al., 2018) see supplement for details and Supplementary Tables S1, S2 for compositional details.

### Enzymes

The expression, purification and characterization of C1FaeA1 (JF826027, subfamily 5 (SF5, Dilokpimol et al., 2016), C1FaeA2 (JF826028, SF5), and C1FaeB2 (JF826029, SF6) from *Chrysosporium lucknowense* C1 have been described previously (Kühnel et al., 2012). The expression and production of the FAEs in *Pichia pastoris* X-33 from *Aspergillus niger*: AnFaeB (CAC83933.1, SF1), AnFaeC (XP_001395336.1, SF5), AnFaeA (CAA70510.1, SF7), and AnFaeJ (An15g05280, SF9), from *Aspergillus sydowii*: AsFaeF (Aspsy1_293049, SF1), AsFaeC (Aspsy1_154482, SF5), AsFaeE (Aspsy1_1158585, SF6), and AsFaeI (Aspsy1_160668, SF13), from *Aspergillus nidulans*: AnidFAEC (AN5267, SF5), from *Fusarium oxysporum*: FoFae2 (Fusox1_8990, SF6), and *Stereum hirsutum*: ShFae1 (Stehi1_73641, SF13) has previously been described (Dilokpimol et al., 2018) (Table 1). The *P. pastoris* X-33 harboring the corresponding genes were grown according to Dilokpimol et al. (2017). The induction was continued for 96 h at 22°C with 0.5% (v/v) methanol supplement every 24 h. Culture supernatants were harvested (4000 × g, 4°C, 20 min), filtered (0.22 μm; Merck Millipore, Darmstadt, Germany) and stored at −20°C prior to further analysis.

### Enzyme Filtering and Measurement of Protein Content

Approximately 7.5 mL of the culture filtrates containing the individual FAE and the culture filtrate without FAE (broth) were concentrated to approx. 2 mL using ultrafiltration (Amicon Ultra, molecular mass cut-off of 3 kDa, Merck Millipore, Cork, Ireland). The filtrate was removed, and the concentrate of the culture filtrates containing the individual FAE and the culture filtrate without FAE diluted with a sodium acetate buffer (50 mM, pH 5.8) to 7 mL and concentrated again using ultrafiltration. This washing procedure was performed twice. The protein content of the enzyme-containing concentrates and the broth from *P. pastoris* without FAE insertion (negative control, coded as “broth”) were measured using a BCA Protein Assay Kit (Thermo Scientific, Rockford, IL, United States). The results from the broth was compared to the results obtained from a BSA Protein Assay Kit – Reducing agent compatibility (Thermo Scientific, Rockford, IL, United States), where similar protein concentration was measured as described above. For both assays bovine serum albumin (BSA) was used as a standard. After protein content determination, all enzymes were diluted to a final protein concentration of 1 mg/mL in a sodium acetate buffer (50 mM, pH 5.8).

### Incubation of FAEs With Synthetic Model Substrates

Activity assays toward the model substrates methyl ferulate and methyl *p*-coumarate were performed in 200 μL reaction mixtures. 2 mM substrate was dissolved in a sodium acetate buffer (50 mM, pH 5.8) (175 μL) and 25 μg of FAE-containing filtrate (1 mg/mL) or the broth (1 mg/mL) was added. In contrast, 1 μg of the purified FAEs from *Chrysosporium lucknowense* C1 (C1FaeA1, C1FaeA2, and C1FaeB2) was used for the activity assays. The reaction mixtures were incubated at 37°C for 2 and 19 h. All reactions were performed in duplicate. At 2 and 19 h, 50 μL was taken from the reaction mixture and diluted 10 times with MilliQ. All reactions were stopped by incubating the samples at 99°C for 2 min. Samples were stored in the freezer and thawed shortly before subjection to RP-UHPLC-UV-ESI-MS/MS.

### Determination of Free and Bound HCAs in PCW-Derived and Natural Substrates

The maximum release (free and bound content) of HCAs in the substrates was estimated based on the amounts released in 0.5 M KOH (saponification). In total, 5 mg of substrate (or 1 mg for CSlignin), 500 μL of acetate buffer (or 100 μL for CSlignin; 50 mM, pH 5.8) and 500 μL (or 100 μL for CSlignin) of 0.5 M KOH were mixed. Incubation were prepared in triplicate. The free amount of HCAs in the substrates was determined by mixing 5 mg of substrate (or 1 mg for CSlignin), and 500 μL of acetate buffer (or 100 μL for CSlignin; 50 mM, pH 5.8). This preparation was prepared in duplicate. All samples were incubated for 19 h in the dark at 37°C, under head-over-tail mixing. After incubation, saponified samples were acidified (pH ∼3) by addition of 6 M HCl. All samples were centrifuged (12,000 *g*, 10 min, 4°C), and appropriately (between 10 and 50 times) diluted before RP-UHPLC-UV-ESI-MS/MS analysis.

### Enzyme Activity Assays With PCW-Derived and Natural Substrates

Enzyme activity assays toward PCW-derived and natural substrates were performed for all 14 FAEs and the broth. The insoluble substrates (SBP, CS, and WS) were weighed in the corresponding amounts. CFoligo was dissolved in acetate buffer (50 mM, pH 5.8) to a stock solution of 10 mg/mL. CSlignin was dissolved in EtOH/CHCl_3_ 50:50 to 1 mg/mL and after distribution of sample-material the solvent was evaporated under a stream of nitrogen. A lower amount of CSlignin was used due to the limited substrate availability. The reactions were performed in the presence of 5 mg of substrate (or 1 mg of CSlignin) in 500 μL (or 100 μL for CSlignin) of a sodium acetate buffer (50 mM, pH 5.8). Enzyme loading was 25 μg for FAE-containing filtrates (1 mg/mL), and 2.5 μg enzyme for purified FAEs from *C. lucknowense* C1 (C1FaeA1, C1FaeA2, and C1FaeB2). All reactions were performed in duplicate. The samples were incubated for 19 h in the dark at 37°C, with head-over-tail mixing. Enzymes were inactivated at 99°C for 5 min. All samples were centrifuged (12,000 *g*, 10 min, 4°C), and appropriately diluted before RP-UHPLC-UV-ESI-MS/MS analysis. The released amount of HCAs by the enzymes was determined as the total sum of HCAs subtracted by the free amount (section Enzymes) present. Percentages are presented based on total esterified contents (section Enzymes).

#### RP-UHPLC-UV-ESI-MS/MS Analysis: Model Substrates Methyl Ferulate and Methyl *p*-Coumarate Incubated With FAEs

All samples were analyzed on an Accela reversed phase ultra-high performance liquid chromatography (RP-UHPLC) system, equipped with a pump, degasser, autosampler, and photodiode array (PDA) detector (Thermo Scientific, San Jose, CA, United States). Samples (5 μL) were injected onto an Acquity UPLC BEH C18 column (150 × 2.1 mm, particle size 1.7 μm) (Waters, Milford, MA, United States). To ensure the stability of the released compounds, the temperature of the autosampler was kept at 4°C during the analysis. The flow rate was 400 μL/min at 45°C. The binary mobile phases consisted of (A) water + 0.1% formic acid and (B) acetonitrile + 0.1% formic acid. The elution profile was as follows: Isocratic on 5% B; 0.0–1.5 min, B linearly from 5 to 60%; 1.5–20.0 min, B linearly from 60 to 100% B; 20.0–20.1 min, isocratic on 100% B; 20.1–25.0 min, B linearly from 100 to 5% B; 25.0–26.0 min, isocratic on 5% B; 26.0–31.0 min. UV spectra for methyl *p*-coumarate and methyl ferulate were recorded at 310 and 320 nm, respectively. No MS data was acquired for the incubation of the model substrates with the FAEs. The decrease in substrate concentration representing activity was determined from a standard curve of the substrates (0.625–50 μg/mL). Data were processed using Xcalibur 2.2 (Thermo Fisher Scientific).

#### RP-UHPLC-UV-ESI-MS/MS Analysis: HCA Content and Released Amounts From PCW-Derived and Natural Substrates

Samples were analyzed by using RP-UHPLC-UV as described above with a modified elution profile combined with electrospray ionization – ion trap mass spectrometry (ESI-ITMS) detection. To ensure the stability of the released compounds, the temperature of the autosampler was kept at 4°C during the analysis. The binary mobile phases consisted of (A) water + 0.1% formic acid and (B) acetonitrile + 0.1% formic acid. The elution profile was as follows: The first 2 min isocratic on 5% B; 0.0–2.0 min, B linearly from 5 to 40%; 2.0–13.0 min, B linearly from 40 to 100% B; 13.0–13.1 min, isocratic on 100% B; 13.1–18.0 min, B linearly from 100 to 5% B; 18.0–18.1 min, isocratic on 5% B; 18.1–23.0 min. The flow rate was 0.400 mL/min. UV spectra of FA and *p*CA were recorded at 310 and 285 nm, respectively. MS data was acquired by using an LTQ-Velos Pro mass spectrometer (Thermo Fisher Scientific) equipped with a heated ESI probe. Nitrogen was used as sheath gas and auxiliary gas. Data was collected over a m/z range of 120–1500 in negative (NI) mode. Data dependent MS^2^ analysis was performed using collision-induced dissociation with a normalized collision energy of 35%. The ion transfer tube temperature was 300°C, source heater temperature was 250°C and the source voltage was 3.5 kV. Data were processed using Xcalibur 2.2 (Thermo Fisher Scientific). A linear correlation between the MS signal and the concentration of FA and *p*CA was found. Based on the MS molar response factor obtained for FA, the molar response factors of diFAs and triFAs were estimated, as no standards are commercially available. Our values obtained for amounts of diFAs and triFAs based on the estimated molar response factors are expected to be close to actual absolute amounts, but remain to be confirmed. Nevertheless, the incubation of the substrates with FAEs led to a difference in released amounts of diFAs and triFAs, which allowed us to determine variations in the ability of FAEs to release HCA derivatives. For SBP and WAX-i the UV-signal was used, as low amounts of the analyzed compounds were released. The contents of diFAs and triFAs were calculated relative to FA including mass correction. FA and *p*CA standard curves showed linearity both in UV and in MS (data not shown). The MS standard curves for FA and *p*CA resulted in the *R*^2^-values between 0.9723 and 0.9997 for all experiments.

## Results

### Specificity of FAEs Toward Synthetic Model Substrates

**FIGURE 2:**
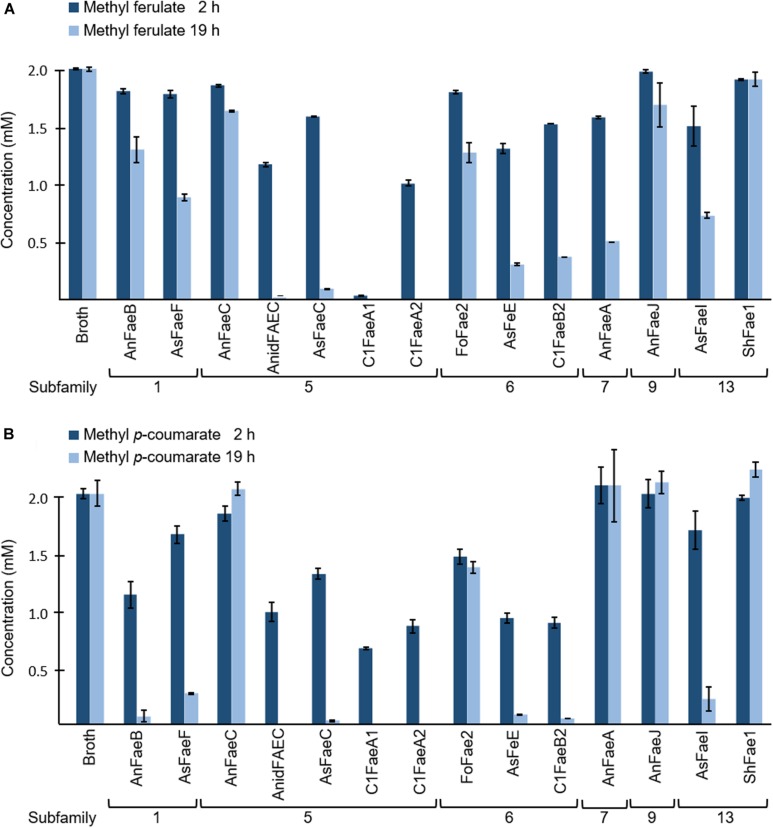
Specificity of FAEs toward **(A)** methyl ferulate (MF) and **(B)** methyl *p*-coumarate (MpC). Both methyl ferulate (2 mM) and methyl *p*-coumarate (2 mM) were incubated with and without (broth) FAEs at 37°C for 2 and 19 h. The reduction in methyl ferulate and methyl *p*-coumarate concentration was measured in duplicate using UHPLC-UV (*n* = 2). The broth is the culture supernatant of *P. pastoris*, which was grown without FAE insertion (negative control). Error bars represent the average standard deviation based on determined absolute numbers.

Usually, the specificity of FAEs is assayed via synthetic substrate conversion. Therefore, we assayed the specificity of our 14 FAEs toward the two synthetic substrates methyl ferulate and methyl *p*-coumarate after 2 and 19 h of incubation (Figure 2 and Supplementary Table S3). These two substrates were chosen from the four most commonly used model substrates (methyl ferulate, methyl *p*-coumarate, methyl caffeate, and methyl sinapate), as FA and *p*CA are the major HCAs present in PCWs (Crepin et al., 2004). Thirteen out of 14 FAEs that were tested for their activity toward methyl ferulate decreased its concentration after an incubation time of 2 h (Figure 2A). Only ShFae1 (SF13) did not decrease the methyl ferulate concentration within 2 h. After 19 h, the methyl ferulate concentration decreased further for the other 13 tested FAEs. Among them, the FAEs of SF5 almost completely converted methyl ferulate after 19 h, except AnFaeC (Figure 2A). The incubation of methyl *p*-coumarate with FAEs also resulted in a decrease in the methyl *p*-coumarate concentration (Figure 2B). In brief, nine FAEs showed activity toward methyl *p*-coumarate after 2 h of incubation. For those FAEs, almost all methyl *p*-coumarate had been hydrolyzed after 19 h. In contrast, the incubation of methyl *p*-coumarate with AnFaeC (SF5), AnFaeA (SF7), AnFaeJ (SF9), and ShFae1 (SF13) did not lead to a decrease in the substrate concentration, even after an incubation time of 19 h. AnFaeC and ShFAE1 showed a higher activity toward methyl *p*-coumarate within the first 2 h compared to the 19 h incubation, although both enzymes showed only a relatively low activity toward this substrate. The incubation of methyl ferulate and methyl *p*-coumarate with the broth from a *P. pastoris* strain, which did not contain any of the selected FAEs, did not alter the methyl ferulate and methyl *p*-coumarate concentration after 2 or 19 h (Figures 2A,B).

### Identification of HCAs in PCW-Derived and Natural Samples

In total, four compounds were chosen as representatives of different classes of PCW-derived substrates: SBP is a pectin isolate, soluble CFoligo are composed of branched xylo-oligosaccharides, which are highly feruoylated (Appeldoorn et al., 2013), WAX-i is an insoluble xylan (Harris and Trethewey, 2010), and CSlignin is a HCA-rich lignin isolate (Supplementary Files S5, S6 and Supplementary Tables S1, S2). In addition, the natural substrates CS and WS were used as representatives of HCA-rich lignocellulose, mainly composed of xylan, lignin and cellulose (van Dongen et al., 2011; Bakker et al., 2013). The total carbohydrate content and composition of all PCW-derived and natural substrates is shown in Supplementary Files S5, S6 and Supplementary Tables S1, S2.

The presence of various types of esterified HCAs was analyzed for all PCW-derived and natural substrates through saponification. RP-UHPLC-UV-ESI-MS/MS showed the release of FA, *p*CA, eight different diFAs of which three are proposed diFAs (m/z 389, m/z 401_9.74 min_, m/z 401_10.66 min_), and two triFAs under alkaline conditions (0.5 M KOH, Table 2). Tentative structures of diFAs were proposed based on MS^2^ data (Table 2 and Supplementary Figures S2, S3). In addition, four diFAs (8-8′-aryl-diFA, 8-5′-diFA, 5-5′-diFA, and 8-*O*-4′-diFA) were identified based on retention time using reversed phase separation, which corresponded to previous reported data (Table 2 and Supplementary Figures S2C,D,F,H; Appeldoorn et al., 2010; Kühnel et al., 2012; Vismeh et al., 2013).

**TABLE 2:** Esterified diferulic (diFAs) and triferulic (triFAs) acids analyzed by RP-UHPLC-UV-ESI-MS/MS.

Compound	Rt (min)	Theoretical mass	Observed mass [M-H]^–†^	MS^2^ product ions (relative intensity)^‡^
8-8′-(furan)-diFA	8.23	404.11	403	134 (7.8), 151 (7.2), 165 (7.1), 178 (7.5), 193 (100), 209 (5.0), 215 (12.9), 341 (59.9)
m/z 389_8.22 min_	8.22	390.13	389	151 (5.8), 165 (7.0), 178 (6.4), 181 (7.8), 193 (100), 195 (16.0), 321 (30), 341 96.9), 343 (13.0)
8-8′-(aryl)-diFA	8.36	386.10	385	341 (100)
8-5′-diFA	8.72	386.10	385	297 (29.6), 341 (100)
m/z 401_9.74 min_	9.74	402.10	401	325 (8.0), 357 (100.0)
5-5′-diFA	10.03	386.10	385	282 (6.9), 326 (11.6), 341 (100), 342 (9.7), 370 (13.5)
m/z 401_10.66 min_	10.66	402.10	401	191 (25.3), 235 (9.1), 357 (30.9), 371 (34.5), 383 (100)
8-*O*-4′-diFA	11.00	386.10	385	193 (100), 249 (8.1), 313 (70.4), 317 (7.4), 341 (79.1)
triFA 1^§^	11.34	578.14	577	311 (6.5), 355 (34.2), 489 (5.7), 533 (100.0), 534 (6.2)

*Tentative structures have been proposed based on their retention time and MS^2^ fragmentation pattern (Figure 1 and Supplementary Figure S2; Appeldoorn et al., 2010; Kühnel et al., 2012; Vismeh et al., 2013). ^†^[M-H]^–^refer to the observed mass peak representing the negative ions; loss of 1 Da. ^‡^Intensity: the highest peak correspond to 100%, the other peaks are given as percentages hereof. Only peaks which were higher than 5% in the intensity are reported. ^§^TriFA 2_12.38 min_ is not shown, as no MS^2^ data was obtained for this compound.*

We propose the identification of 8-8′-(furan)-diFA (CFoligo, 8.23 min, m/z 404; Table 2 and Supplementary Figure S2A), in addition to the previously reported diFAs (8-8′-aryl-diFA, 8-5′-diFA, 5-5′-diFA, and 8-*O*-4′-diFA). The presence of the 8-*O*-4′-diFA has been reported for PCWs and in particular for CFoligo (Bunzel et al., 2006; Schatz et al., 2006; Appeldoorn et al., 2010). Interestingly, a peak (R_*t*_ 8.23 min) in the UV chromatogram was observed for saponified SBP, WAX-i, CS, WS, and CSlignin, and corresponded to the predominant MS peak comprising a m/z 389 (390 Da; Supplementary Figure S2B). Based on the co-elution with 8-8′-(furan)-diFA and the main MS^2^ fragment (m/z 393; Supplementary Figure S2A and Table 2), it is plausible that this compound is related to 8-8′-(furan)-diFA.

Further, two structurally unknown diFAs with a m/z 401 were observed (402 Da, R_*t*_ 9.74 and 10.66 min, respectively) (Table 2 and Supplementary Figures S2E,G). Although these diFAs have been previously observed, their structure has not yet been elucidated (Appeldoorn et al., 2010; Kühnel et al., 2012). Based on the mass, it is likely that both compounds are dehydrodimers composed of FA and 5-hydroxyferulic acid, which are also involved in HCA biosynthesis (Humphreys et al., 1999; Morreel et al., 2004). Furthermore, we observed that the diFAs m/z 401_10.66 min_ was unstable during saponification (Tables 2, 3 and Supplementary Figure S2G). Finally, only one triFA was identified (R_*t*_ 11.35 min, Table 2 and Supplementary Figure S3).

**TABLE 3:** Determined (free and bound) hydroxycinnamic acids in PCW-derived and natural substrates.

Compound	Type	SBP^†^ (μg/mg sample)	CFoligo (μg/mg sample)	WAX-i^†^ (μg/mg sample)	CS (μg/mg Sample)	WS (μg/mg sample)	CSlignin (μg/mg sample)
FA^‡^	Free	0.05 ± 0.01	74.5 ± 1.6	0.02 ± 0.00	0.13 ± 0.00	0.06 ± 0.00	0.01 ± 0.00
	Bound	15.0 ± 0.3	57.3 ± 1.2	15.0 ± 0.2	12.7 ± 0.6	5.51 ± 0.09	8.82 ± 0.16
*p*CA^‡^	Free	0.04 ± 0.00	5.58 ± 0.20	0.04 ± 0.00	1.13 ± 0.01	0.39 ± 0.00	0.78 ± 0.01
	Bound	0.21 ± 0.00	6.54 ± 0.12	0.98 ± 0.04	28.4 ± 1.4	6.60 ± 0.16	98.3 ± 2.8
m/z 389_8.22 min_	Free	0.02 ± 0.00	1.08 ± 0.12^§^	0.01 ± 0.00	0.46 ± 0.05	1.01 ± 0.08	0.03 ± 0.00
	Bound	0.60 ± 0.12	20.0 ± 0.4^§^	0.51 ± 0.03	27.9 ± 1.0	30.3 ± 0.6	45.9 ± 2.2
8-8′-aryl-diFA	Free	n.d.	n.d.	n.d.	n.d.	n.d.	n.d.
	Bound	n.d.	45.1 ± 2.3	1.13 ± 0.04	3.39 ± 0.23	0.99 ± 0.05	2.37 ± 0.17
8-5′-diFA	Free	n.d.	4.13 ± 0.15	n.d.	n.d.	n.d.	n.d.
	Bound	0.23 ± 0.01	56.6 ± 1.2	1.13 ± 0.04	13.8 ± 0.7	2.45 ± 0.09	5.45 ± 0.23
m/z 401_9.74 min_	Free	n.d.	7.10 ± 0.03	n.d.	0.20 ± 0.00	n.d.	n.d.
	Bound	n.d.	19.9 ± 0.4	n.d.	5.54 ± 0.08	0.97 ± 0.02	9.31 ± 0.04
5-5′-diFA	Free	n.d.	15.6 ± 1.0	n.d.	n.d.	n.d.	n.d.
	Bound	0.21 ± 0.00	154 ± 4	2.58 ± 0.07	8.35 ± 0.27	2.66 ± 0.09	1.29 ± 0.13
m/z 401_10.66 min_^¶^	Free	n.d.	5.83 ± 0.25	n.d.	n.d.	0.17 ± 0.00	n.d.
	Bound	0.04 ± 0.00	*n*/*a*	0.18 ± 0.00	0.32 ± 0.01	0.16 ± 0.02	n.d.
8-O-4′-diFA	Free	n.d.	3.55 ± 0.10	n.d.	n.d.	n.d.	n.d.
	Bound	0.54 ± 0.02	106 ± 3	2.97 ± 0.00	6.00 ± 0.16	2.54 ± 0.19	n.d.
TriFA 1_11.35 min_	Free	n.d.	0.64 ± 0.10	n.d.	n.d.	n.d.	n.d.
	Bound	0.06 ± 0.00	26.8 ± 0.9	2.16 ± 0.06	n.d.	n.d.	n.d.
TriFA 2_12.38 min_	Free	n.d.	n.d.	n.d.	n.d.	n.d.	n.d.
	Bound	0.05 ± 0.00	13.9 ± 0.7	2.22 ± 0.06	n.d.	n.d.	n.d.
Total	Free	0.11	118	0.07	1.92	1.63	0.82
	Bound	17.0	505	28.9	106	52.2	171
Total (%) w/w	Free	0.01	11.8	0.01	0.19	0.16	0.08
	Bound	1.70	50.5	2.89	10.6	5.22	17.1

*Structural details of the analyzed HCAs (mono, di-, and triFAs) are presented above (Figure 1, Supplementary Figures S2, S3, and Table 2). Numbers of the quantified (FA, pCA) and estimated free and bound (total – free amount) HCAs content is presented in μg/mg sample (dry matter). ^†^Estimated by UV. ^‡^Estimated by UV. Correlation to MS determination confirmed (Supplementary Figure S1). ^§^ The m/z 389 was not observed, however, the major mass peak with m/z 403 corresponds to the proposed 8-8 **′**-(furan)-diFA. ^¶^The measured bound amount was less than what has been released when the substrate was incubated with FAEs (see later results). Consequently, this compound is rendered unstable under the basic conditions (saponification at 0.5 M KOH) used for measurement of the total amounts. See M&M for details. CFoligo, corn fiber oligomers; CS, corn stover; CSlignin, corn stover lignin isolate; diFA, diferulic acid; FA, ferulic acid; n/a, not applicable; n.d, not detected; pCA, p-coumaric acid; SBP, sugar beet pectin; triFA, triferulic acid; WAX-i, insoluble wheat arabinoxylans; WS, wheat straw.*

### Analysis of the Free and Bound Content of HCAs

The amount of ester-linked HCAs that were released after saponification (0.5 M KOH) was set to 100%, corresponding to the highest possible release of HCAs (Table 3). Determination of these HCAs for all substrates was performed based on MS, except for SBP and WAX-i. The HCA-content of the latter two were determined based on UV (see M&M for details). Since di- and tri-FA standards were not available, HCAs were estimated based on calibration with “corrected” FA and *p*CA standards (see M&M for details).

The amount of FA, *p*CA, diFAs, and triFAs that were present in the PCW-derived and natural substrates differed vastly (Table 3). As an example, CFoligo was highly feruloylated with FA, diFAs, and triFAs, whereas neither CS, WS, nor CSlignin contained triFAs. The amounts of esterified FA and *p*CA corresponded well with what has been described previously (Micard et al., 1996; Saulnier et al., 2007; Appeldoorn et al., 2013; Damásio et al., 2013). The estimated total amount of HCAs present in SBP, CFoligo, WAX-i, CS, WS, and CSlignin summed up to 1.7, 62.3, 2.9, 10.8, 5.4, and 17.2% w/w (sum of % free and % bound), respectively, based on dry matter (Table 3).

The characterization above shows that the chosen PCW-derived and natural substrates provide a wide range of HCAs that are bound to different structural moieties. Hence, these substrates were subjected to our FAEs in order to further explore the specificity of FAEs from different subfamilies.

### Specificity of FAEs Toward PCW-Derived and Natural Substrates

The specificity of the 14 FAEs toward the PCW-derived and natural substrates was determined by using RP-UHPLC-UV- ESI-MS/MS.

**FIGURE 3:**
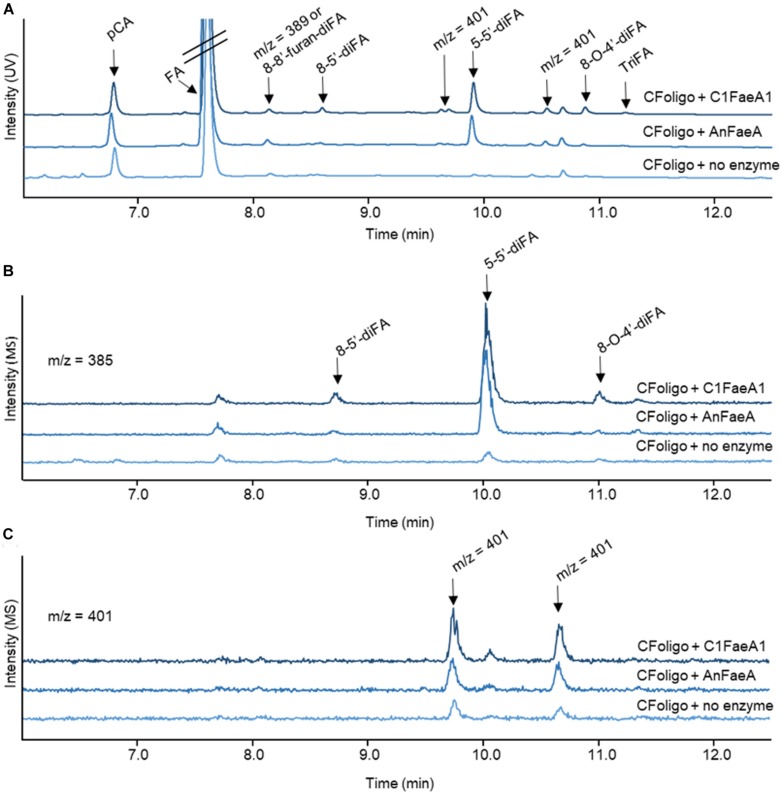
RP-UHPLC-UV-ESI-MS/MS elution profiles for corn fiber oligomers (CFoligo) incubated with FAEs. CFoligo was incubated with either C1FaeA1 or AnFaeA, and without FAE. **(A)** Elution profile based on UV (310 nm) showing the release of ferulic acid (FA); *p*-coumaric acid (*p*CA), diferulic acids (diFAs) and triferulic acids (triFAs) from CFoligo after the incubation with FAEs. **(B,C)** Elution profile of diferulic acids (diFAs) corresponding to the m/z 385 and 401, respectively. The m/z values represent the mass loss of 1 Da (MS operation in negative mode).

As an example, the reversed-phase elution profiles of CFoligo prior to and after incubation with two different FAEs is shown in Figure 3. This figure shows that from CFoligo, FA was most abundantly present in the CFoligo sample before and after incubation with C1FaeA1 and AnFaeA. Furthermore, the release of several diFAs was observed, of which the most pronounced was 5-5′-diFA.

**FIGURE 4:**
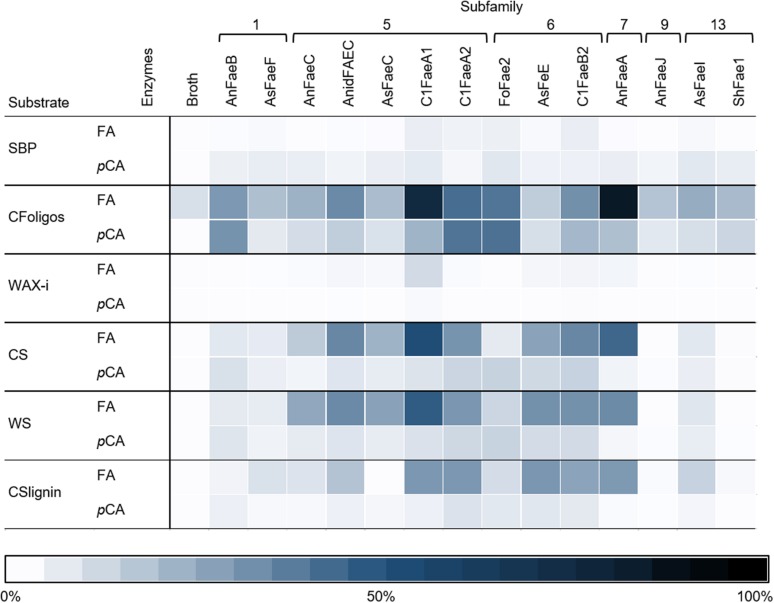
The heatmap shows the specificity of FAEs (representing 6 SFs) toward six substrates. Corn fiber oligomers (CFoligo), corn stover (CS), corn stover lignin isolate (CSlignin), sugar beet pectin (SBP), insoluble wheat arabinoxylans (WAX-i), and wheat straw (WS) were incubated with FAEs and without (broth) at 37°C for 19 h and the release of FA and pCA was measured by RP-UHPLC-UV (*n* = 2). The release of FA and pCA from the substrates by the FAEs are given as percentages of the total amount of ester bound FA and pCA that have been determined by saponification (0.5 M KOH). The broth is the culture supernatant of *P. pastoris* which was grown without FAE insertion (negative control).

Both FA and *p*CA were released from the incubations of CFoligo, CS, WS, and CSlignin with all 14 FAEs (Figure 4 and Supplementary Table S4). Based on maximum relative amounts (100%) of ester bound FA or *p*CA in the tested substrates, FA is released to a larger extent than *p*CA by all FAEs (Supplementary Table S4). However, the proportions of these detected compounds was rather different when absolute amounts of released FA and *p*CA were considered (Supplementary Table S4). For the primary aim of this work, only relative released amounts were considered. Overall, FAEs from SF5, SF6, and SF7 released higher amounts of FA and *p*CA than FAEs from SF1, SF9, and SF13 under the present conditions (Figure 4). As for the model substrates mentioned above, the incubation of all substrates with the control *P. pastoris* fermentation broth did not lead to a substantial release of FA or *p*CA (Figure 4 and Supplementary Table S4).

**FIGURE 5:**
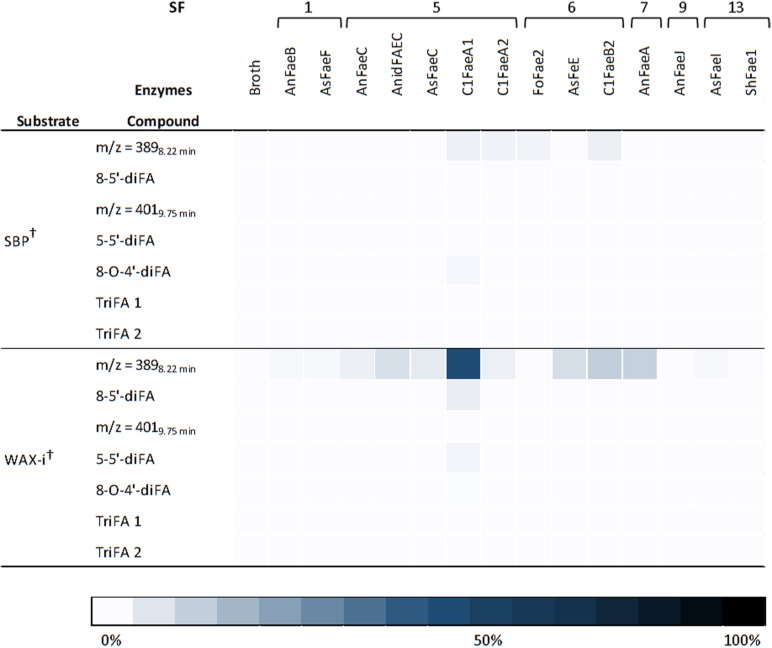
Heatmap presenting the FAE activity toward diFAs and triFAs from incubations with SBP and WAX-i. Substrates were incubated with the 14 FAEs and the fermentation broth for 19 h at 37°C in duplicates. The results are gives as percentages of bound content as measured by RP-UHPLC-UV (*n* = 2). Abbreviations: diFA, diferulic acid; SBP, sugar beet pectin; triFA, triferulic acid; WAX-i, insoluble wheat arabinoxylans. The broth is the culture supernatant of Pichia pastoris which was grown without FAE insertion (negative control). Error bars represent the average standard deviation based on determined absolute numbers. ^†^Estimated by UV. See M&M for details.

**FIGURE 6:**
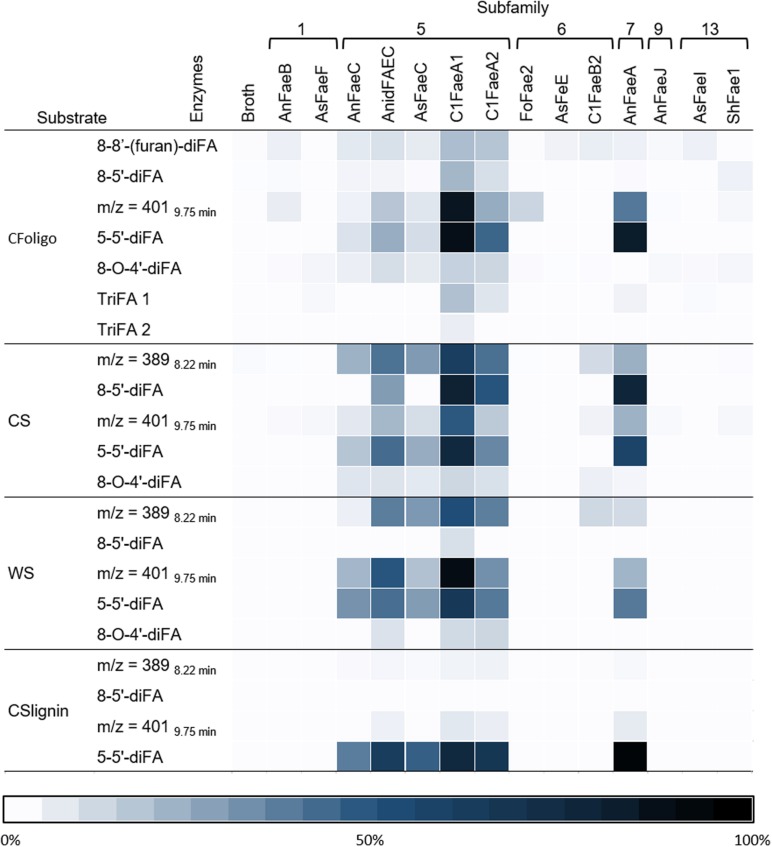
Heatmap presenting the specificity of FAEs. This heatmap highlights the release of diFAs and triFAs by 14 FAEs (representing 6 SFs) from the PCW-derived and natural substrates corn fiber oligomers (CFoligo), corn stover (CS), wheat straw (WS), and corn stover lignin isolate (CSlignin). All substrates were incubated with FAEs and without (broth) at 37°C for 19 h in duplicate. The results are given as percentages of bound content as measured by RP-UHPLC-UV-ESI-MS/MS (*n* = 2). The broth is the culture supernatant of *P. pastoris* which was grown without FAE insertion (negative control).

In addition to FA and *p*CA, most of the FAEs released various diFAs and triFAs, especially from CFoligo, CSlignin, CS, and WS (Figures 5, 6 and Supplementary Table S3). A clear release of diFAs was only observed for the FAEs classified as SF5 and SF7. FAEs of the other SFs did not release diFAs from these substrates. In contrast, the incubation of SBP and WAX-i with the FAEs released either none or very low amounts of the various diFAs and triFAs (Figure 5 and Supplementary Table S5).

**FIGURE 7:**
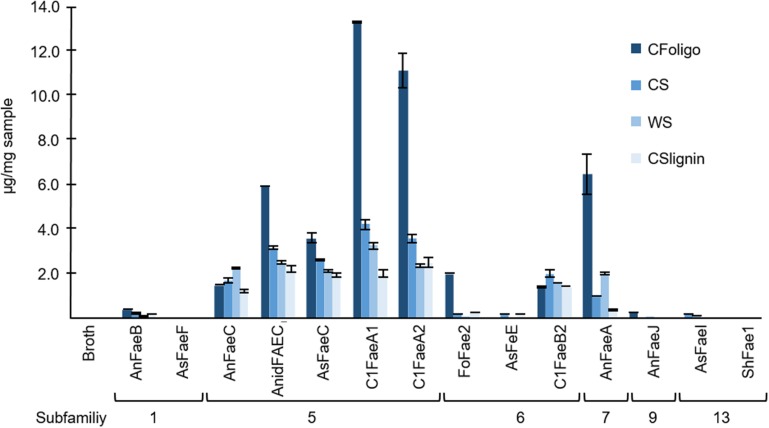
The release of the diFA corresponding to a m/z value of 401_10.66 min_. The release of this diFA (μg of compound/mg dry matter of natural substrate) from corn fiber oligomers (CFoligo), corn stover (CS), wheat straw (WS), and corn stover lignin isolate (CSlignin) that were incubated with and without (broth) FAEs at 37°C for 19 h. Samples were measured by RP-UHPLC-UV-ESI-MS/MS (*n* = 2). The broth is the culture supernatant of *P. pastoris* which was grown without FAE insertion (negative control). Error bars represent the average standard deviation based on determined absolute numbers.

The diFA composing a m/z value of 401_10.66 min_ was unstable during saponification (0.5 M KOH). Therefore, the total amount of the diFA (m/z 401_10.66 min_) could not be determined and the relative release of this diFA from the substrates incubated with FAEs was not calculated. As a result, the released amounts of diFA m/z 401_10.66 min_ are presented separately in Figure 7 (Supplementary Table S6). Apart from CFoligo, this diFA was released in similar amounts by FAEs of SF5, SF7, and C1FaeB2 of SF6, whereas this diFA was either not or barely released by FAEs of SF1, 9, and 13.

## Discussion

### Fungal FAEs From Different SFs

In this study, we determined the specificity of 14 fungal FAEs from six different SFs via the analysis of the release of different HCAs from two model substrates and six PCW-derived and natural substrates (Dilokpimol et al., 2016). It is expected that these results provide further insights in the predictability of the SF classification for substrate specificities of FAEs, which eases the selection of suitable FAE candidates for industrial applications.

Notably, some FAE family members, especially of SF 5 and 6, have also been described to release acetic acid from acetyl-esterified model substrates (Table 1). In this study, the release of acetyl residues from PCW-derived and natural substrates has not been further investigated. Three FAEs (C1FaeA1, C1FaeA2, and C1FaeB2) have previously been purified and intensely studied by Kühnel et al. (2012). These FAEs were chosen because they enlarge SF5 and 6, and their activity toward a variety of substrates have been described earlier. The other expressed FAEs that were used in this study were pre-purified by filtration from the culture supernatant.

It is important to note that we mainly point out differences between the substrate specificities of FAEs from different SF toward the tested substrates, rather than specify the exact amounts of HCAs released (Table 4). All incubations were performed with the same protein concentration and it is likely that the amount of FAEs, which were present in the protein fractions varied due to differences in the expression efficiency. Moreover, all incubations have been performed at the same conditions (37°C and pH 5.8). These conditions were closely related to optimal pH, temperature, and stability of most of these FAEs reported in previous works (Table 1; Kühnel et al., 2012; Dilokpimol et al., 2016). Thus, the absolute catalytic performance of the FAEs that was determined based on the release of HCAs from various substrates might be higher at the optimal pH condition. Still, variations in the specificity of these FAEs among these substrates, like methyl ferulate and methyl *p*-coumarate, and the release of structural-different HCAs from the six PCW-derived and natural substrates can be determined. Finally, the representation of SF7 and SF9 has to be critically evaluated, since only one FAE of SF7 and one of SF9 was investigated in this study (Table 1). To indisputably define the substrate specificity of these SFs, more SF9 and SF13 candidates need to be studied.

**TABLE 4:** Overview of the HCA-release from the incubation of synthetic model substrates, plant cell wall-derived substrates, including lignin, and natural substrates with FAE of various SFs.

		Subfamily					
Substrates	HCA	1	5	6	7	9	13
MF	MF^a^	++	± | +++	++| +++	++	±	± | ++
M*p*C	M*p*C^a^	+++	± | +++	++| +++	±	±	± | +++
SBP	FA	±	±	±	±	±	±
	*p*CA	±	±	±	±	±	±
	DiFAs	±	±	±	±	±	±
	TriFAs	±	±	±	±	±	±
CFoligos	FA	± | ++	± | ++	± | ++	+++	±	± | ++
	*p*CA	± | ++	± | ++	± | ++	±	±	±
	DiFAs	±	± | ++ | +++	±	± | ++ | +++	±	±
	TriFAs	±	±	±	±	±	±
WAX-i	FA	±	±	±	±	±	±
	*p*CA	±	±	±	±	±	±
	DiFAs	±	± | ++	±	±	±	±
	TriFAs	±	±	±	±	±	±
CS	FA	±	± | ++	± | ++	++	±	±
	*p*CA	±	±	±	±	±	±
	DiFAs	±	± | ++	±	± | ++	±	±
	TriFAs	n.a.^b^	n.a.^b^	n.a.^b^	n.a.^b^	n.a.^b^	n.a.^b^
WS	FA	±	± | ++	± | ++	++	±	±
	*p*CA	±	±	±	±	±	±
	DiFAs	±	± | ++ | +++	±	± | ++	±	±
	TriFAs	n.a.^b^	n.a.^b^	n.a.^b^	n.a.^b^	n.a.^b^	n.a.^b^
CSlignin	FA	±	± | ++	± | ++	++	±	±
	*p*CA	±	±	±	±	±	±
	DiFAs	±	± | ++	±	± | +++	±	±
	TriFAs	n.a.^b^	n.a.^b^	n.a.^b^	n.a.^b^	n.a.^b^	n.a.^b^

*The symbols represent percentages which are calculated based on the release of HCAs (FA, pCA, diFAs and triFAs) from substrates incubated with FAEs of one SF compared to the total amount of ester bond HCAs that have been determined by saponification of the substrates. The type of HCA and the ratio within these compounds released varied for different FAEs of one SF (Figures 4–6), which leads to the occurrence of more than one symbol. The substrate specificity is shown as low (“±”; 0–20%), medium (“++”; 20–80%) and/or high (“+++”; 20–80%) values. ^a^Results represent the incubation of MF and MpC with FAEs for 19 h (Figure 4). ^b^No release of TriFA was determined from the incubation of these substrates with FAEs.*

### Activity of FAEs Toward Synthetic Model Substrates

First, the enzyme activity of FAEs toward two synthetic FAE substrates (methyl ferulate and methyl *p*-coumarate) was compared. Among all FAEs, SF5 FAEs showed the highest activity toward both model substrates (Table 4). Moreover, SF1 FAEs (AnFaeB and AsFaeF) were moderately active toward methyl ferulate, but showed a strong activity toward methyl *p*-coumarate, which is in line with previously reported data (Figure 2 and Table 1; Crepin et al., 2004; Dilokpimol et al., 2017, 2018; Antonopoulou et al., 2018). SF6 FAEs has previously been shown to cleave both methyl ferulate and methyl *p*-coumarate, which is agreement with our findings (Figure 2 and Table 1; Kühnel et al., 2012; Dilokpimol et al., 2018). Interestingly, AnFaeA of SF7 showed a high specificity toward methyl ferulate, where 75% had been degraded after 19 h, but was inactive toward methyl *p*-coumarate. A previous study has shown that the Tyr80 of this AnFaeA interacts with the methoxyl group at the C3 position of FA (Hermoso et al., 2004; McAuley et al., 2004; Faulds et al., 2005). An absence of this methoxyl group may reduce the substrate binding, which could possibly explain why AnFaeA did not cleave methyl *p*-coumarate. Surprisingly, AnFaeA did release *p*CA from PCW-derived and natural substrates (Figures 2, 4). How the different structural properties of these substrates influence the substrate binding of AnFaeA is currently unknown. AsFaeJ (SF9) and ShFae1 (SF13) did not cleave methyl ferulate nor methyl *p*-coumarate, whereas AsFaeI (SF13) was active toward both methyl ferulate and methyl *p*-coumarate (Figure 2). Apparently, SF13 FAEs vary in their substrate specificity toward these model substrates, as the SF13 FAEs *Um*ChlE from *Ustilago maydis* showed activity toward both methyl ferulate and methyl *p*-coumarate, what is similar to the AsFaeI (Nieter et al., 2015).

### Activity of FAEs Toward Six PCW-Derived and Natural Substrates

Next, the specificity for releasing various HCAs of the 14 FAEs toward six PCW-derived and natural substrates was determined. Generally, the FAEs exhibited a low, but still detectable specificity toward SBP and WAX-i (Figures 4, 5 and Table 4). These findings match those of previous studies, which showed that FAEs comprise a low specificity toward branched and highly substituted substrates, like SBP and WAX-i. The incubation of these substrates with hydrolases, like xylanases or pectinases, will lead to a cleavage of the backbone chain and, thereby, an enhanced solubility of the substrates (Crepin et al., 2004; Kühnel et al., 2012; Kumar et al., 2013; Mäkelä et al., 2018). Some of the FAEs tested might comprise activity toward acetyl esters, which could have decreased the stearic hindrance and improved the binding toward the substrates tested (Tenkanen et al., 1991; Hashimoto et al., 2010).

Incubations of the natural substrates CS and WS with FAEs demonstrated that FAEs of SF5, SF6, and SF7 released the same types of HCAs from these substrates as observed for the fully soluble CFoligo (Figures 4, 6 and Table 4). The physical pretreatment (ball-milling) that was applied to the substrates enhanced the accessibility and also partly dissolved the cell walls of CS (26.6 ± 0.9% w/w dry matter) and WS (17.8 ± 1.1% w/w dry matter). It can, however, still be argued that FAEs were also active toward the insoluble fraction of CS and WS, since, for example, C1FaeA1 released 64–92% of various diFAs, such as 8-5′-diFA, 5-5′-diFA, and m/z 401_9.75 min_ (Figure 4 and Supplementary Table S5) and it is unlikely that these HCAs originated entirely from the soluble fraction. The latter is, however, not further confirmed in this work.

The two tested SF1 FAEs showed a low release (<20%) of FA and *p*CA from the xylan-type substrates (CS, WS, and CSlignin) and a moderate release of both FA and *p*CA was observed from soluble CFoligo (Figures 4, 6, Table 4, and Supplementary Table S4). Moreover, SF1 FAEs set free only very low amounts of diFAs and triFAs from the xylan-type substrates, whereas higher amounts of these compounds were released from soluble CFoligo. While SF13 FAEs exhibited an almost similar substrate specificity toward these substrates as SF1 FAEs, their subdivision is justified based on their different specificity toward, in particular, methyl *p*-coumarate (Figure 2 and Table 4; Nieter et al., 2015; Dilokpimol et al., 2016). The latter is also reflected in the relatively higher absolute amounts of *p*CA than FA released from CFoligo by SF1 compared to SF13 FAEs (Supplementary Table S4). Similar to SF1 and SF13, the SF9 FAE did not release any HCAs from the PCW-derived and natural substrates tested, except for CFoligo (Figure 6). Moreover, no *p*CA was released from CFoligo by this SF9 FAE, which indicates that this FAE is distinctive from SF1 and SF13 FAEs. The inactivity of this SF9 enzyme might coincide with its similarity to other SF9 tannase-like members, which are not active toward these types of ester-linked HCAs and, possibly, should not be considered true FAEs (Dilokpimol et al., 2016; Antonopoulou et al., 2018). However, possible conclusions in relation to SF9 FAEs are limited, as only one member has been investigated in this study.

FAEs of SF5 and SF6, released FA and *p*CA from most PCW-derived and natural substrates (CFoligo, CS, WS, and CSlignin) (Figure 4 and Table 4). In contrast, SF7 released FA from these substrates, but was only able to release *p*CA from the soluble CFoligo (Figure 4). In addition, the FAEs from SF5 and SF7 released higher amounts of diFAs and triFAs, compared to all other FAEs tested. AnFaeC (SF5) released diFAs from CFoligo, CS, WS, and CSlignin, while previous research has indicated that this FAE did not release diFAs from PCW-derived and natural substrates, such as WAX-i and SBP (Dilokpimol et al., 2017). Most likely, the absence of released diFAs from WAX-i and SBP by FAEs relates to the low amounts of diFAs that are present in these substrates (Table 2). SF6 FAEs hardly released diFAs and triFAs, except for the incubation of CS with C1FaeB2 (Figures 5, 6). The general inability to release diFAs from PCW-derived and natural substrates of the SF6 FAEs is in accordance with previous data, which reported SF6 FAEs as type B FAEs which are incapable to release diFAs from PCW materials (Table 1; Crepin et al., 2004; Dilokpimol et al., 2016). Interestingly, in comparison to the SF6 enzyme AsFaeE, the incubation of CFoligo with the SF6 enzyme FoFae2 showed the highest specificity for FA and *p*CA, while for the synthetic substrates, CS, WS, and CSlignin, AsFaeE released higher levels of FA and *p*CA compared to FoFae2 (Figure 4). These results indicate mostly the variation in substrate specificity amongst FAEs of the same SF, which likely results from variations of the amino acids that are present in the proximity of the substrate-binding site and the catalytic pocket of the FAEs (Hermoso et al., 2004; McAuley et al., 2004; Faulds et al., 2005; Goldstone et al., 2009; Uraji et al., 2018).

Remarkably, SF5 enzymes were ranked as different types according to the ABCD classification, such as type A, C, or C/D, which does not reflect their overall ability to release *p*CA, FA, diFAs, and triFAs from both synthetic as well as PCW-derived and natural substrates (Table 1; Debeire et al., 2012; Kühnel et al., 2012; Dilokpimol et al., 2017, 2018). Based on our results, the co-classification of these FAEs in SF5 shows that the SF classification covers the FAE promiscuity and reflects their common evolutionary origin to a larger extent than the ABCD classification.

In particular, FAEs of SF5, SF6, and SF7 released higher amounts of FA from PCW-derived and natural substrates, compared to *p*CA (Figure 4), even though rather high amounts of esterified *p*CA were present in these substrates (i.e., 28.39 and 98.25 μg/mg sample for CS and CSlignin, respectively) (Ralph, 2010; Reinoso et al., 2018).

The type of structure of diFAs released by FAEs of SF5 and SF7 from various substrates differed. Generally, the release of 5-5′-diFA was most prevalent from CFoligo, CS, and WS (up to 91% for CFoligo), followed by m/z 389_8.22 min_ (up to 61% for CS) and m/z 401_9.75 min_ (up to 87 and 92% for CFoligo and WS, respectively). Of the released diFAs from CSlignin by SF5 and SF6 FAEs, 5-5′-diFA was most common (up to 97%), while the m/z 389_8.22 min_, which is the most predominant diFA of this substrate (45.93 μg/mg CSlignin), was hardly released. These differences might again be ascribed to the complexity of the lignin structure (Ralph, 2010). Furthermore, it is tempting to speculate that certain types of diFAs preferentially cross-link between the arabinoxylan chains rather than cross-linking with lignin, which might steer FAE specificity. The 8-*O*-4′ diFA was hardly released from any substrate, not even from CFoligo in which relatively high amounts of this structure were present (153.83 μg/mg sample for CFoligo). This suggests that the 8-*O*-4′ diFA structure is less accessible for FAEs compared to other diFAs, like the 5-5′-diFA (Figure 1), which might either be the result of the catalytic site structure of the FAEs or of substrate inaccessibility.

### Release of FA and *p*CA From Lignin by FAEs

For the first time we show that several FAEs, such as C1FaeA1, C1FaeA2, and AnFaeA, were able to release *p*CA from lignin (isolated from CS), next to the release of FA and diFAs as mentioned above (Tables 3, 4). Although *p*CA has been shown to be γ-esterified to lignin, ester linkages of FA and diFAs were not identified in the 2D-NMR spectrum nor in literature (Supplementary File 7). But, since FA-like HCAs (FA, diFAs and triFAs) have been shown to be esterified to arabinofuranosyl residues (Ara), we have calculated the molar ratio of FA-like HCAs to Ara. Saponification of 1 mg CSlignin led to a release of 0.81 μmole of FA, diFAs, and triFAs (FA-derived HCAs). In comparison, 0.07 μmole arabinofuranosyl residues were determined to be present in CSlignin, which corresponds to a molar ratio of approximately 12:1 (FA-derived HCAs:arabinofuranosyl residues). These results support that a part of the released FA-derived HCAs could be ester-linked to lignin in corn stover (van Erven et al., 2017). For the three FAEs (C1FaeA1, C1FaeA2, and AnFaeA) that showed the highest activity toward CSlignin, between 0.02 and 0.07 μmole of FA-derived HCAs from 1 mg of CSlignin were released resulting in a molar ratio ranging from 0.3:1.0 up to 0.75:1.0 (FA-derived HCAs:arabinofuranosyl residues) (Table 2). Thus, all FA and diFAs released by these FAEs could theoretically have originated from highly substituted Ara residues in CSlignin. Since the lignin isolate was thoroughly water-washed, it is expected that the remaining oligosaccharides were bound to lignin (covalent or non-covalent) (van Erven et al., 2017). Though the release of lignin-linked FA-derived HCAs by FAEs can thus not be concluded and needs to be investigated in future work, at the very least, our results signify that HCAs can be released from lignin-bound carbohydrate-HCA complexes.

## Conclusion

FAE specificity is highly relevant for industrial applications and it is important to evaluate this specificity on PCW-derived and natural substrates rather than synthetic model substrates alone. We demonstrate that FAEs show a vast variation in their ability to hydrolyze HCAs from PCW-derived and natural substrates. Furthermore, the type of HCA and the ratio within these compounds released varied for different FAEs and substrates. In addition to FA and *p*CA, the 5-5′ diFA structure was released most by the FAEs tested, while the 8-*O*-4′- counterpart was released to a much lower extent.

Both substrate specificity and product profiles of the FAEs tested were linked to the new SF classification. This study supports the reliability of the SF classification, which recognizes a more subtle difference in FAE specificity, compared to the ABCD classification. Based on this study, FAEs of SF5 and SF7 showed the highest release of FA, *p*CA, and diFAs over the range of substrates, while FAEs of SF6 were comparable but less pronounced for diFAs release (Table 4). These results suggest that SF5 and SF7 FAEs are promising enzymes for biorefinery applications, like the production of biofuels, where a complete degradation of the plant cell wall is desired. In contrast, SF6 FAEs might be of interest for industrial applications that require a high release of only FA and *p*CA, which are needed as precursors for the production of biochemicals. Therefore, our work provides new insights on the selection of suitable FAE-candidates for industrial applications.

## Data Availability Statement

All datasets generated for this study are included in the article/Supplementary Material.

## Author Contributions

EU, MF, RV, and MK designed the research. EU and MF performed the research and analyzed the data from FAE studies. GE performed isolation and analysis of the CSlignin substrate. AD produced the FAE filtrates. EU, MF, and MK wrote the manuscript. AD, GE, and RV critically revised the manuscript. All authors read and approved the final manuscript.

## Conflict of Interest

The authors declare that the research was conducted in the absence of any commercial or financial relationships that could be construed as a potential conflict of interest.

## Abbreviations

Ara: arabinofuranosyl residue
8-8′-(aryl)-diFA: 8-8′-(aryl)-diferulic acid
BSA: bovine serum albumin, CFoligo, corn fiber oligomers
CS: corn stover
CSlignin: corn stover diFA diferulic acid
diFAs diferulic acids: lignin isolate
8-*O*-4′-diFA: 8-*O*-4′-diferulic acid
5-5′-diFA: 5-5′-diferulic acid
8-5′-diFA: 8-5′-diferulic acid
diFA: diferulic acid, FA: ferulic acid
FAE: feruloyl esterase
8-8′-(furan)-diFA: 8-8′-(tetrahydrofuran)-dehydroferulic acid
G: guaiacyl subunit
H: *p*-hydroxyphenyl subunit
HCA: hydroxycinnamic acid
MF: methyl ferulate
MpC: methyl *p*-coumarate
MS: mass spectrometry
NI: negative mode
*p*CA: *p*-coumaric acid
PCW: plant cell wall
S: syringyl subunit
SBP: sugar beet pectin
SF: subfamily
triFA: triferulic acid
triFAs: triferulic acids
WAX-i: wheat arabinoxylans insoluble
WS: wheat straw.
