# Supplementary material for: Feruloyl Esterases for Biorefineries: Subfamily Classified Specificity for Natural Substrates
Source: Front Bioeng Biotechnol. 2020 Apr 23;8:332. doi: 10.3389/fbioe.2020.00332 (PMC7191039; doi:10.3389/fbioe.2020.00332)
Supplement: Supplementary file 1 [file Data_Sheet_1.PDF]

Supplementary material

Feruloyl esterases for biorefineries: subfamily classified specificity for natural substrates

Emilie N. Underlin[1,2 †], Matthias Frommhagen[1 †], Adiphol Dilokpimol[3], Gijs van Erven[1], Ronald P. de Vries[3], Mirjam A. Kabel[1,a]

[†] These authors contributed equally to this work.

[1] Laboratory of Food Chemistry, Wageningen University & Research, Wageningen, The Netherlands

[2] Technical University of Denmark, Lyngby, Denmark

[3] Fungal Physiology, Westerdijk Fungal Biodiversity Institute and Fungal Molecular Physiology, Utrecht University, Utrecht, The Netherlands

[a] **Correspondence**

Mirjam A Kabel

mirjam.kabel@wur.nl

Supplementary material

Supplementary File	Figure/Table	Title	Page
**1**	Figure S1	Comparison of the possible release in percentages of FA (A) and *p*CA (B) from CFoligo from both UV and MS measurements, showing similarity	**3**
**2**	Figure S2	MS^2 fragmentation spectra and tentative structures of identified diferulic acids (diFAs).	**4**
**3**	Figure S3	Mass fragmentation (MS^2) spectrum of $triFA_{11.35\ min}$	**5**
**4**	Table S1	Carbohydrate content and composition of PCW-derived and natural substrates used to determine the substrate specificity of FAEs	**6**
**5**	Table S2	2D HSQC NMR spectroscopy analysis of CSlignin<br>Semi-quantitative HSQC NMR structural characterization of CS lignin isolate<br>Short discussion of carbohydrate content and composition and lignin composition of PCW-derived and natural substrates	**7-8**
**6**	Table S3	Numerical values for the measured substrate concentrations (mM) and the standard deviations of MF and MpC after 2 and 19 h	**9**
**7**	Table S4	The release and standard deviation of FA and pCA as percentages of the bound content of all PCW-derived and natural substrates [µg/mg sample]	**10**
**8**	Table S5	The release and standard deviation of diFAs and triFAs as percentages of the bound content of all PCW-derived and natural substrates [µg/mg sample]	**11-13**
**9**	Table S6	Exact values for the release of m/z = $401_{10.66\ min}$	**14**
		References	**15**

a)

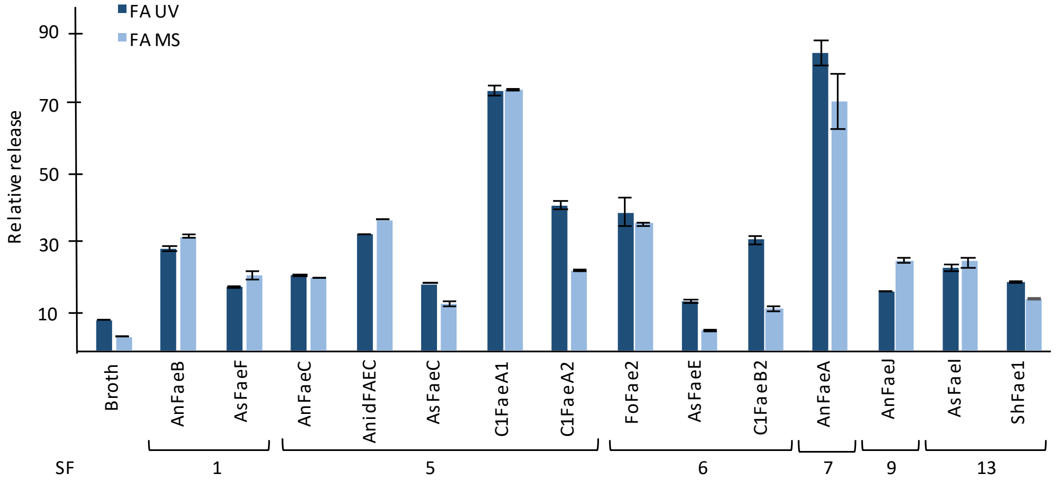

b)

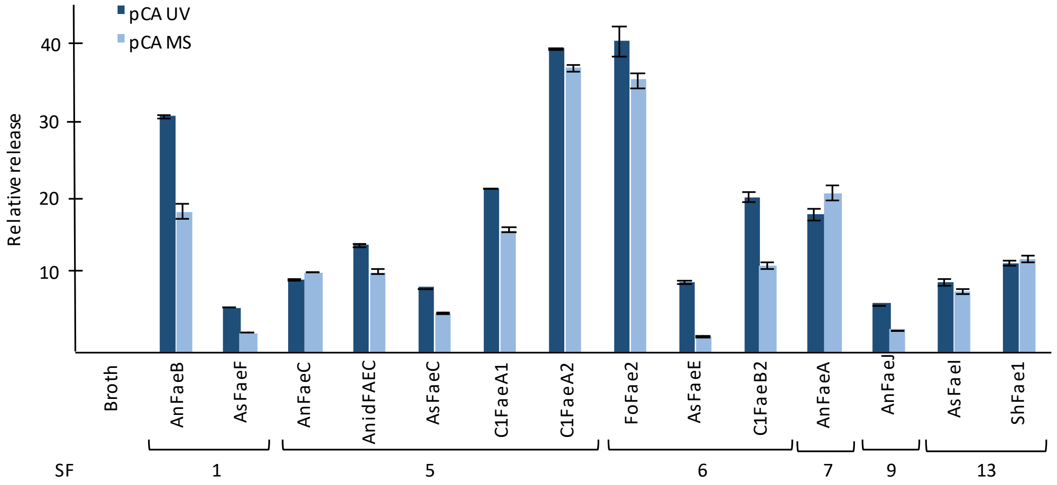

Supplementary File 1: Figure S1. Comparison of the relative release in % of FA (a) and pCA (b) from CFoligo from both UV and MS measurements (n = 2). The broth is the culture supernatant of *Pichia pastoris* which was grown without FAE insertion (negative control). Error bars represent the average standard deviation based on determined absolute numbers.

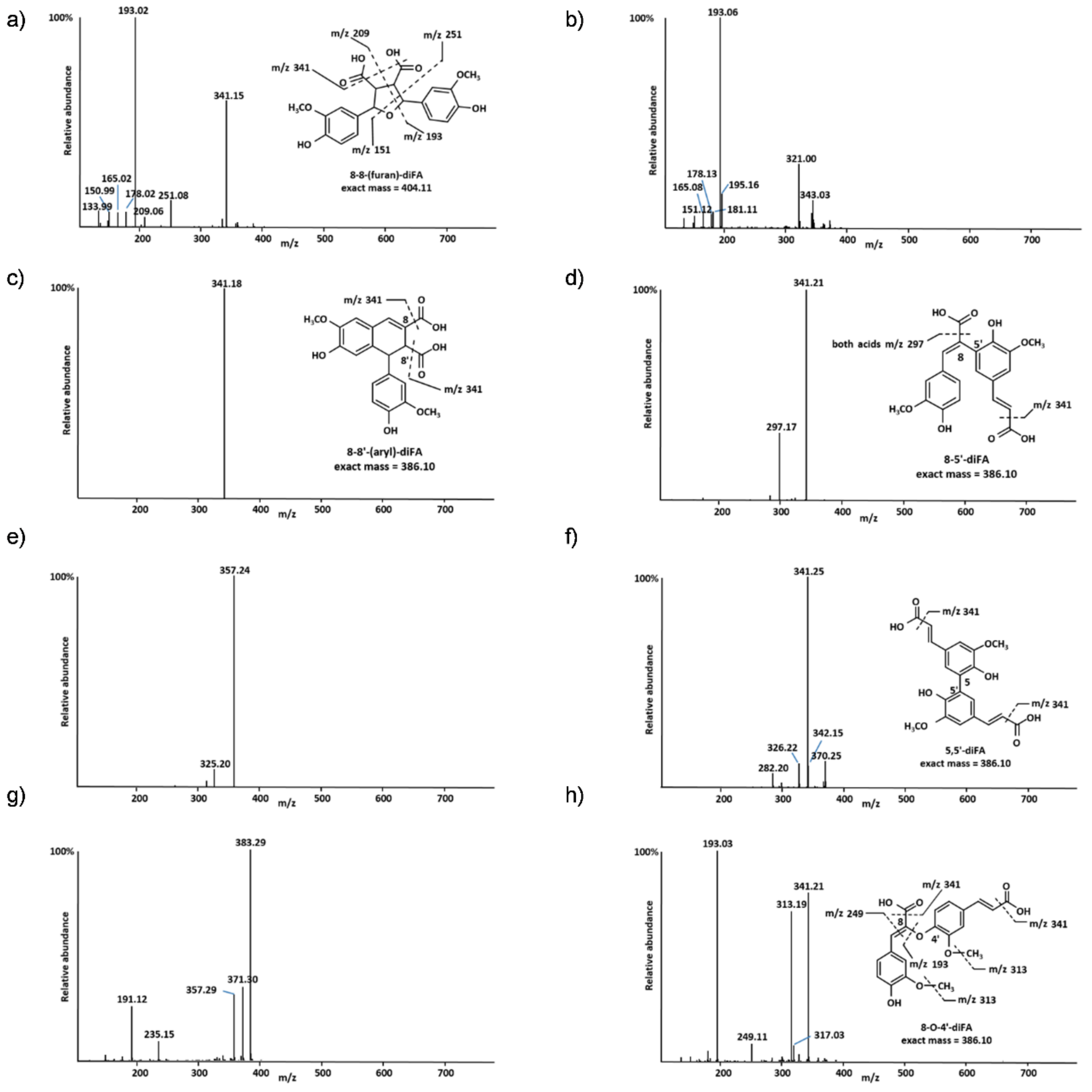

Supplementary File 2: Figure S2. MS^2 fragmentation spectra and tentative structures of identified diferulic acids (diFAs). a) 8-8'-(furan)-diFA; b) m/z 389 $_{8.22\ min}$; c) 8-8'-(aryl)-diFA; d) 8-5'-diFA; e) diFA (m/z 4019.75 min), likely, a covalently linked ferulic acid (FA) and 5-hydroxyferulic acid (m/z 357; loss of carboxylic acid); f) 5-5'-diFA; g) diFA (m/z 401 $_{10.66\ min}$), likely a covalently linked FA and 5-hydroxyferulic acid (m/z 357; loss of carboxylic acid, m/z 371; loss of OCH_3); h) 8-O-4'-diFA. Tentative structures have been proposed based on MS^2 and retention time (Figure 1, Table 1). See main manuscript for further details.

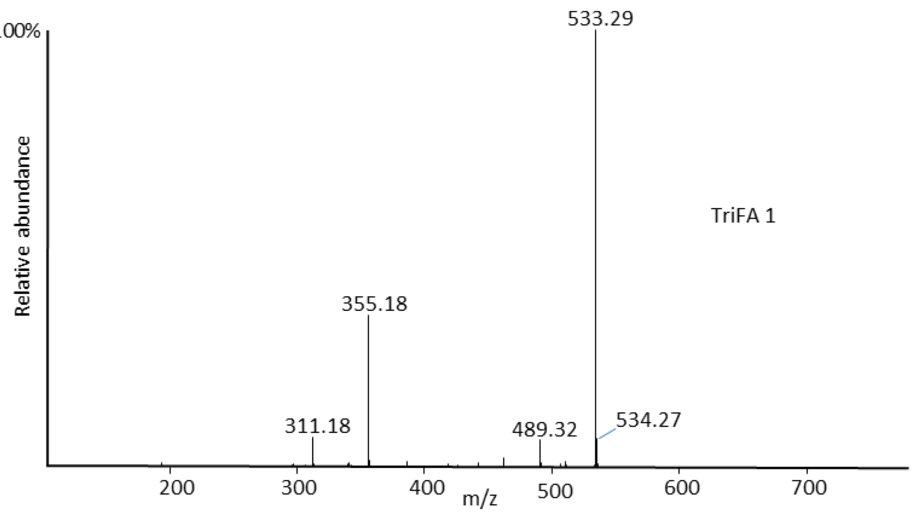

Supplementary File 3: Figure S3. Mass fragmentation (MS^2) spectrum of triFA$_{11.35\ min}$.

u

Supplementary File 4:

Details on analysis of neutral sugar content and composition and uronic acid content

The neutral sugar content and composition of sugar beet pectin (SBP) and insoluble wheat arabinoxylan (WAX-i) was determined in duplicate according to Englyst and Cummings (1984) using inositol as an internal standard. Samples were treated with 72% (w/w) H_2SO_4 (1 h, 30°C) followed by hydrolysis with 1 M H_2SO_4 for 3 h at 100 °C and the constituent sugars released were analyzed as their alditol acetates using gas chromatography (Focus-GC, ThermoScientific, Waltham, MA, USA). The column used was DB-225 (15 m × 0.53 mm i.d. × 1 µm film thickness; Agilent Technologies, Santa Clara, CA, USA). The initial column temperature was 180 °C with 2 min holding time. The temperature was then increased to 210 °C with a ramp of 2 °C/min, followed by 5 min holding time. The injector and detector temperature was set at 220 °C. Helium was used as carrier gas with a constant pressure of 60 kPa. The analyses were performed in duplicate. Total carbohydrate content was calculated as the sum of neutral carbohydrates and uronic acids.

The uronic acid content of SBP and WAX-i was determined in duplicate as anhydro-uronic acid content by an automated m-hydroxydiphenyl assay (Thibault 1979) with addition of sodium tetraborate using an autoanalyser (Skalar Analytical BV, Breda, The Netherlands). Glucuronic acid (Fluka AG, Busch, Switzerland) was used as a reference (12.5–200 µg/mL).

Table S1. Carbohydrate content and composition of PCW-derived and natural substrates used to determines the substrate specificity of FAEs

	Carbohydrate content % (w/w) based on dry matter							
**Name**	**Xyl**	**Ara**	**Gal**	**Glc**	**Rha**	**Man**	**Uronic acids**	**Total**
SBP†	n.d.	5.00 ± 0.00	9.00 ± 0.00	1.00 ± 0.00	1.00 ± 0.00	n.d.	53.5 ± 0.71	69.0 ± 1.41
CFoligo‡	25	8	3	6	0	0.3	2.0	45
WAX-i†	36.5 ± 0.71	23.0 ± 0.00	1.00 ± 0.00	16.0 ± 0.00	n.d.	3.00 ± 0.00	3.00 ± 0.00	82.5 ± 0.71
CS§	40.0 ± 0.42	4.90 ± 0.19	1.49 ± 0.04	49.3 ± 0.56	n.d.	n.d.	4.28 ± 0.10	63.53 ± 1.20
WS§	37.4 ± 0.05	3.84 ± 0.00	0.97 ± 0.01	53.2 ± 0.26	n.d.	n.d.	4.22 ± 0.19	64.72 ± 2.26
CSlignin§	5.25 ± 0.00	0.90 ± 0.00	0.32 ± 0.01	1.30 ± 0.00	n.d.	n.d.	1.00 ± 0.00	8.8 ± 0.00

Sugar beet pectin (SBP), corn fiber oligosaccharides (CFoligo), insoluble arabinoxylans (WAX-i), ball milled corn stover (CS), and wheat straw (WS), and corn stover lignin isolate (CSlignin)

† The determination of the neutral sugar and uronic acid content is described above

‡, § Carbohydrate analysis and composition as published previously in Appeldoorn et al. (2013) and van Erven et al. (2017), respectively.

Supplementary File 5:

2D HSQC NMR spectroscopy analysis of CSlignin

For structural characterization of the corn stover lignin isolate by NMR, around 16 mg of the isolate was swollen in 0.6 mL DMSO-d_6. 2D heteronuclear single quantum coherence (HSQC) NMR was performed according to previously described methods (Kim, Ralph, and Akiyama 2008; Mansfield et al. 2012; del Río et al. 2012; van Erven et al. 2018). The spectra were recorded at 25 °C with Bruker's standard pulse sequence "hsqcetgpsisp2.2" on a Bruker AVANCE III 600 MHz NMR spectrometer (Bruker BioSpin, Rheinstetten, Germany) equipped with a 5 mm cryo-probe located at MAGNEFY (MAGNEtic resonance facilitY, Wageningen, The Netherlands). The spectral widths were 7,200 and 30,000 Hz for the ^{1}H and ^{13}C dimensions, respectively. The number of collected complex points was 2018 in the ^{1}H dimension with a relaxation time of 1 s. The number of collected scans was 16 and 400 increments of time were recorded in the ^{13}C dimension. The $^1J_{CH}$ used was 145 Hz. The data were processed with Bruker TopSpin 3.5 software. Processing used Gaussian apodization in ^{1}H and a squared cosine function in the ^{13}C dimension. For ^{13}C, data were zero filled up to 1024 points prior to Fourier transformation. The solvent peak (DMSO-d_6) was used as an internal reference (δ_C 39.5 ppm; δ_H 2.49 ppm). HSQC correlation peaks were assigned by comparison with literature (Kim et al. 2008; Mansfield et al. 2012; del Río et al. 2012, 2015; Ralph et al. 2009). Semi-quantitative analysis of the volume integrals was performed according to Del Río *et al.* (del Río et al. 2012). Alternatively, in the aliphatic oxygenated region *β*-*O*-4' substructures were estimated from their C_β-H_β correlations, since they were shown to be interfered to a lesser extent by the presence of carbohydrates (Mansfield et al. 2012). For phenylcoumaran and tetrahydrofuran substructures, their respective C_α-H_α correlations were used. Volume integrals for tetrahydrofuran substructures were logically halved. $S_{2,6}$, G_2 and $H_{2,6}$ signals were used for S, G and H units, respectively, where S and H integrals were halved as well. Oxidized analogues were estimated in a similar manner. Tricin, *p*CA and FA were similarly estimated from their respective $T_{2',6'}$, pCA$_{2,6}$ and FA$_2$ signals. $H_{2,6}$ integrals were corrected for the overlapping phenylalanine cross-peak (PHE$_{3,5}$) by subtraction of the isolated PHE$_{2,6}$ cross-peak (Kim et al. 2017). The extent of γ-acylation of *β*-*O*-4' substructures was estimated from the respective C_γ-H_γ correlations. Volume integration was performed at equal contour levels.

Table S2. Semi-quantitative HSQC NMR structural characterization of CS lignin isolate

	**CS lignin**
**Lignin subunits (%)**†	
**H**	1
**G**	55
**S (S_{ox})**	43 (0.3)
**S/G**	0.8
**Hydroxycinnamates (%)**‡	
***p*-coumarate**	68
**ferulate**	10
***p*-CA/FA**	7
**Lignan (%)**‡	
**tricin**	8
**Lignin end-groups (%)**‡	
***γ*-acylated cinnamyl alcohol**	1
**Lignin inter-unit linkages (%)**§	
***β-O*-4' aryl ethers (*γ*-acylated)**	90 (48)
***β*-5' phenylcoumarans**	3
***β-β* tetrahydrofurans (*γ*-acylated)**	7 (7)
**total**	100

† Percentage of subunits (H+G+S+S_{ox} =100)

‡ Percentage versus subunits

§Percentage of total inter-unit linkages with the amount of *γ*-acylated analogues in parentheses. Extent of *γ*-acylation might be slightly overestimated due to overlap with carbohydrate cross-peaks

Short discussion of carbohydrate content and composition and lignin composition of PCW-derived and natural substrates

As expected, CFoligo, WAX-i, CS, and WS were found to be rich in arabinoxylans (xylopyranosyl groups 25 – 40% (w/w), arabinofuranosyl groups 3-23% (w/w) and glucuronic acid 2-4% (w/w)), while SBP consisted mainly of galacturonic acids (53% (w/w)). The HSQC NMR analysis confirmed that the obtained CSlignin fraction is representative for a typical grass lignin (Supplementary Table S2) (del Río et al. 2012; Mansfield, Kim, Lu, & Ralph 2012). The obtained CSlignin isolate was of high purity, and comprised only a low carbohydrate content (8.8% (w/w)). HSQC NMR analysis of CSlignin revealed typical structural features of grass-type lignin (Supplementary Table S2). In detail, CSlignin largely consisted of guaiacyl (G) and syringyl (S) subunits, which were found to be mainly linked via *β-O*-4' aryl ethers (90%). Large amounts of *p*CA and FA subunits (68% and 10%, for *p*CA and FA, respectively) were found to be incorporated in the isolated lignin. All *p*CA was considered to be *γ*-acylated to the inter-unit linkages of CSlignin (Lu and Ralph 2005). About half of all *β-O*-4' aryl ethers was *γ*-acylated in CSlignin. Furthermore, the extensive inclusion of *γ*-acyl esters also caused the formation of either single- or double-acylated *β-β* tetrahydrofuran linkages (Supplementary Table S2).

Supplementary File 6: Table S3. Numerical values for the measured substrate concentrations (mM) and the standard deviations of MF and MpC after 2 and 19 h[†]

Enzyme	SF	MF_{2h} (mM)	Std. dev.$_{MF\ 2h}$	MF_{19h} (mM)	Std. dev.$_{MF\ 19h}$	MpC_{2h} (mM)	Std. dev.$_{MpC\ 2h}$	MpC_{19h} (mM)	Std. dev.$_{MpC\ 19h}$
Broth		2.00	0.00	2.00	0.02	2.00	0.04	2.00	0.11
AnFaeB	1	1.80	0.02	1.30	0.11	1.13	0.11	0.07	0.05
AsFaeF	1	1.78	0.03	0.88	0.03	1.65	0.08	0.27	0.01
AnFaeC	5	1.85	0.01	1.64	0.01	1.83	0.06	2.04	0.06
AnidFAEC	5	1.17	0.02	0.01	0.00	0.98	0.09	0.00	0.00
AsFaeC	5	1.59	0.01	0.09	0.00	1.30	0.04	0.03	0.01
C1FaeA1	5	0.03	0.00	0.00	0.00	0.66	0.01	0.00	0.00
C1FaeA2	5	1.00	0.02	0.00	0.00	0.85	0.06	0.00	0.00
FoFae2	6	1.80	0.01	1.27	0.08	1.45	0.06	1.36	0.05
AsFeE	6	1.31	0.04	0.30	0.01	0.92	0.05	0.08	0.01
C1FaeB2	6	1.52	0.00	0.37	0.00	0.88	0.05	0.05	0.00
AnFaeA	7	1.57	0.01	0.50	0.00	2.07	0.16	2.07	0.31
AnFaeJ	9	1.98	0.02	1.69	0.19	2.00	0.12	2.10	0.10
AsFaeI	13	1.50	0.17	0.73	0.02	1.69	0.16	0.22	0.11
ShFae1	13	1.91	0.01	1.91	0.06	1.96	0.02	2.21	0.06

[†] Spectrophotometric methods are most commonly applied to determine the activity of FAEs towards synthetic model substrates (Crepin et al. 2004; Kühnel et al. 2012; Dilokpimol et al. 2016, 2017, 2018). These types of assays are based on the de-esterification of an aromatic methyl ester to the corresponding aromatic acid, which is spectrophotometrically analyzed in a fast and easy way. Even though the spectrophotometric assay is widely used and accepted, there is concern about the spectral overlapping of the substrates and the products as their absorbance maxima are close, such as 321.5 and 310 nm for MF and FA, respectively, and 310 and 285 nm for MpC and *p*CA, respectively (all values λ_{max} at pH 6.0) (Faulds and Williamson 1994; Dilokpimol et al. 2016). Consequently, in our research, we quantified the exact amount of remaining substrate by using RP-UHPLC coupled to UV detection (n = 2). In contrast to the above mentioned spectrophotometric assay, this RP-UHPLC-UV method allows the exact quantification of the remaining MF and MpC, where a reduction in substrate concentration is considered to represent FAE activity (starting concentration was 2 mM). The broth is the culture supernatant of *Pichia pastoris* which was grown without FAE insertion (negative control). Error bars represent the average standard deviation based on determined absolute numbers. See M&M for details.

Supplementary File 7: Table S4. The release and standard deviation of FA and pCA as percentages of the bound content of all PCW-derived and natural substrates [μg/mg sample]

Substrate	Enzymes	Broth	AnFaeB 1	AsFaeF 1	AnFaeC 5	AnidFAEC 5	AsFaeC 5	C1FaeA1 5	C1FaeA2 5	FoFae2 6	AsFaeE 6	C1FaeB2 6	AnFaeA 7	AnFaeJ 9	AsFaeI 13	ShFae1 13
SBP	FA	0.04	0.47	1.12	0.16	0.54	0.35	4.11	3.06	3.73	1.08	4.16	0.35	0.05	1.29	0.06
	Std. dev.$_{FA}$	0.00	0.01	0.04	0.00	0.08	0.00	0.10	0.05	0.04	0.36	0.10	0.01	0.00	0.10	0.00
	*p*CA	0.00	3.72	4.73	4.30	2.78	4.16	5.45	2.01	6.17	3.31	3.57	4.15	2.54	5.90	4.62
	Std. dev.$_{pCA}$	0.00	0.19	0.21	0.05	0.09	0.09	0.07	0.12	0.13	0.09	0.05	0.16	0.08	0.26	0.03
CFoligo	FA	8.43	28.84	17.73	21.34	32.91	18.64	73.37	41.05	39.00	13.89	31.18	84.21	16.23	23.21	19.16
	Std. dev.$_{FA}$	0.02	0.86	0.21	0.49	0.18	0.11	1.54	1.04	3.78	0.16	1.25	3.61	0.01	1.05	0.40
	*p*CA	0.00	30.36	5.55	9.06	13.70	8.03	20.96	39.11	40.01	8.79	19.84	17.67	5.92	8.76	11.34
	Std. dev.$_{pCA}$	0.00	0.25	0.08	0.10	0.10	0.08	0.01	0.21	2.03	0.10	0.67	0.73	0.06	0.47	0.25
WAX-i	FA	0.00	0.22	0.26	0.69	1.74	1.40	9.92	0.82	0.18	2.00	2.42	2.27	0.00	0.26	0.00
	Std. dev.$_{FA}$	0.00	0.01	0.004	0.04	0.41	0.03	0.28	0.02	0.01	0.03	0.14	0.29	0.00	0.01	0.00
	*p*CA	0.03	0.10	0.00	0.04	0.26	0.00	1.12	0.00	0.02	0.19	0.44	0.19	0.00	0.00	0.00
	Std. dev.$_{pCA}$	0.00	0.00	0.00	0.00	0.002	0.00	0.01	0.00	0.00	0.00002	0.01	0.01	0.00	0.00	0.00
CS	FA	0.05	5.92	5.18	14.34	33.89	21.20	50.85	30.14	5.39	25.86	33.64	42.93	0.08	5.99	0.41
	Std. dev.$_{FA}$	0.00	0.02	0.24	0.70	0.03	0.28	1.35	0.75	0.07	0.25	0.65	0.39	0.00	0.09	0.01
	*p*CA	0.14	8.21	3.90	2.51	6.63	4.76	7.45	11.43	12.31	10.22	12.22	2.76	0.46	4.23	0.41
	Std. dev.$_{pCA}$	0.00	0.01	0.25	0.11	0.02	0.04	0.21	0.22	0.10	0.07	0.15	0.06	0.00	0.09	0.01
WS	FA	0.05	5.28	4.84	24.59	32.89	26.02	46.24	29.45	10.96	30.79	30.85	32.61	0.10	6.37	0.10
	Std. dev.$_{FA}$	0.002	0.29	0.17	1.38	0.92	0.46	0.34	0.54	0.25	0.71	0.02	1.18	0.0002	0.02	0.0003
	*p*CA	0.09	6.85	3.05	5.05	6.92	4.96	7.78	10.63	12.05	9.60	9.92	1.87	0.50	4.73	0.24
	Std. dev.$_{pCA}$	0.00	0.32	0.04	0.28	0.17	0.10	0.09	0.27	0.06	0.16	0.20	0.09	0.01	0.09	0.00
CSlignin	FA	0.13	2.67	8.00	7.20	16.41	0.13	29.03	29.09	9.07	29.44	25.48	28.66	0.82	12.22	1.16
	Std. dev.$_{FA}$	0.01	0.22	0.58	0.20	1.42	0.34	0.43	0.37	0.58	1.10	0.11	0.94	0.01	0.33	0.05
	*p*CA	0.17	3.75	1.28	1.21	3.51	2.01	3.45	7.62	5.95	6.26	5.66	0.83	0.25	2.16	0.03
	Std. dev.$_{pCA}$	0.00	0.24	0.01	0.02	0.13	0.12	0.01	0.22	0.09	0.19	0.05	0.03	0.01	0.09	0.00

Supplementary File 8:Table S5. The release and standard deviation of diFAs and triFAs as percentages of the bound content of all PCW-derived and natural substrates [µg/mg sample]

	Enzymes	Broth	AnFaeB	AsFaeF	AnFaeC	AnidFAEC	AsFaeC	C1FaeA1	C1FaeA2	FoFae2	AsFeE	C1FaeB2	AnFaeA	AnFaeJ	AsFaeI	ShFae1
Substrate	Compound		1	1	5	5	5	5	5	6	6	6	7	9	13	13
SBP	8-8’-furan-FA	0.00	0.00	0.00	0.00	**0.22**	0.00	**3.48**	**2.90**	**2.63**	0.00	**3.71**	0.00	0.00	0.00	0.00
	std. dev.$_{\text{8-8'-furan-FA}}$	0.00	0.00	0.00	0.00	**0.00**	0.00	**0.06**	**0.15**	**0.14**	0.00	**0.34**	0.00	0.00	0.00	0.00
	8-5'-FA	0.00	0.00	0.00	0.00	0.00	0.00	0.00	0.00	0.00	0.00	0.00	0.00	0.00	0.00	0.00
	std. dev.$_{\text{8-5'-FA}}$	0.00	0.00	0.00	0.00	0.00	0.00	0.00	0.00	0.00	0.00	0.00	0.00	0.00	0.00	0.00
	m/z = 401$_{\text{9.75 min}}$	0.00	0.00	0.00	0.00	0.00	0.00	0.00	0.00	0.00	0.00	0.00	0.00	0.00	0.00	0.00
	std. dev.$_{\text{m/z 401}}$	0.00	0.00	0.00	0.00	0.00	0.00	0.00	0.00	0.00	0.00	0.00	0.00	0.00	0.00	0.00
	5-5'-FA	0.00	0.00	0.00	0.00	0.00	0.00	**1.48**	0.00	0.00	0.00	0.00	0.00	0.00	0.00	0.00
	std. dev.$_{\text{5-5'-FA}}$	0.00	0.00	0.00	0.00	0.00	0.00	**0.09**	0.00	0.00	0.00	0.00	0.00	0.00	0.00	0.00
	8-O-4'-FA	0.00	0.00	0.00	0.00	0.00	0.00	0.00	0.00	0.00	0.00	0.00	0.00	0.00	0.00	0.00
	std. dev.$_{\text{8-O-4'-FA}}$	0.00	0.00	0.00	0.00	0.00	0.00	0.00	0.00	0.00	0.00	0.00	0.00	0.00	0.00	0.00
	Tri-FA 1	0.00	0.00	0.00	0.00	0.00	0.00	0.00	0.00	0.00	0.00	0.00	0.00	0.00	0.00	0.00
	std. dev.$_{\text{TriFA 1}}$	0.00	0.00	0.00	0.00	0.00	0.00	0.00	0.00	0.00	0.00	0.00	0.00	0.00	0.00	0.00
	Tri-FA 2	0.00	0.00	0.00	0.00	0.00	0.00	0.00	0.00	0.00	0.00	0.00	0.00	0.00	0.00	0.00
	std. dev.$_{\text{TriFA 2}}$	0.00	0.00	0.00	0.00	0.00	0.00	0.00	0.00	0.00	0.00	0.00	0.00	0.00	0.00	0.00
CFoligo	8-8’-furan-FA	**0.40**	**3.79**	0.00	**5.67**	**8.23**	**5.14**	**18.33**	**16.00**	**0.39**	**2.93**	**4.34**	**3.53**	**1.36**	**3.55**	0.00
	std. dev.$_{\text{8-8'-furan-FA}}$	**0.01**	**0.39**	0.00	**0.26**	**0.13**	**0.34**	**0.60**	**1.19**	**0.06**	**0.31**	**0.23**	**0.30**	**0.10**	**0.32**	0.00
	8-5'-FA	**0.28**	**0.73**	0.00	**2.48**	**2.32**	**0.59**	**20.11**	**8.79**	0.00	0.00	0.00	**0.63**	0.00	0.00	**3.27**
	std. dev.$_{\text{8-5'-FA}}$	**0.00**	**0.01**	0.00	**0.13**	**0.05**	**0.00**	**0.38**	**0.38**	0.00	0.00	0.00	**0.00**	0.00	0.00	**0.17**
	m/z = 401$_{\text{9.75 min}}$	0.00	**4.48**	0.00	**3.33**	**15.83**	**6.41**	**87.07**	**23.14**	**11.46**	0.00	0.00	**38.07**	**0.50**	0.00	**1.69**
	std. dev.$_{\text{m/z 401}}$	0.00	**0.06**	0.00	**0.02**	**0.11**	**0.01**	**2.72**	**1.10**	**1.08**	0.00	0.00	**1.90**	**0.01**	0.00	**0.06**
	5-5'-FA	0.00	**0.00**	0.00	**7.57**	**22.84**	**9.11**	**90.77**	**43.54**	0.00	0.00	0.00	**82.35**	0.00	0.00	0.00
	std. dev.$_{\text{5-5'-FA}}$	0.00	**0.00**	0.00	**0.33**	**0.01**	**0.03**	**1.65**	**2.29**	0.00	0.00	0.00	**3.74**	0.00	0.00	0.00
	8-O-4'-FA	0.00	**0.64**	**2.03**	**3.91**	**8.95**	**5.29**	**12.51**	**11.15**	**0.65**	**0.23**	**0.34**	**0.10**	**1.43**	**0.90**	**1.75**
	std. dev.$_{\text{8-O-4'-FA}}$	0.00	**0.01**	**0.05**	**0.22**	**0.48**	**0.45**	**0.03**	**0.01**	**0.11**	**0.01**	**0.00**	**0.01**	**0.08**	**0.07**	**0.00**

	Tri-FA 1	0.00	0.00	**1.35**	0.00	0.00	0.00	**17.34**	**6.74**	0.00	0.00	0.00	**2.90**	0.00	**0.71**	0.00
	std. dev.$_{\text{TriFA 1}}$	0.00	0.00	**0.09**	0.00	0.00	0.00	**0.43**	**1.36**	0.00	0.00	0.00	**0.32**	0.00	**0.20**	0.00
	Tri-FA 2	0.00	0.00	0.00	0.00	0.00	0.00	**4.14**	0.00	0.00	0.00	0.00	0.00	0.00	0.00	0.00
	std. dev.$_{\text{TriFA 2}}$	0.00	0.00	0.00	0.00	0.00	0.00	**0.01**	0.00	0.00	0.00	0.00	0.00	0.00	0.00	0.00
	8-8’-furan-FA	0.00	**1.33**	**1.41**	**3.62**	**8.23**	**5.21**	**51.53**	**3.56**	**0.40**	**8.89**	**13.24**	**12.53**	**0.11**	**1.31**	**0.34**
	std. dev.$_{\text{8-8’-furan-FA}}$	0.00	**0.06**	**0.12**	**0.20**	**2.13**	**0.18**	**13.79**	**0.23**	**0.02**	**0.19**	**0.50**	**0.50**	**0.00**	**0.07**	**0.03**
	8-5'-FA	0.00	0.00	0.00	0.00	0.00	0.00	**4.15**	0.00	0.00	0.00	0.00	0.00	0.00	0.00	0.00
	std. dev.$_{\text{8-5'-FA}}$	0.00	0.00	0.00	0.00	0.00	0.00	**0.23**	0.00	0.00	0.00	0.00	0.00	0.00	0.00	0.00
	m/z = 401$_{\text{9.75 min}}$	0.00	0.00	0.00	0.00	0.00	0.00	0.00	0.00	0.00	0.00	0.00	0.00	0.00	0.00	0.00
	std. dev.$_{\text{m/z 401}}$	0.00	0.00	0.00	0.00	0.00	0.00	0.00	0.00	0.00	0.00	0.00	0.00	0.00	0.00	0.00
WAX-i	5-5'-FA	0.00	0.00	0.00	0.00	**0.15**	0.00	**2.04**	0.00	0.00	0.00	0.00	**0.14**	0.00	0.00	0.00
	std. dev.$_{\text{5-5'-FA}}$	0.00	0.00	0.00	0.00	**0.03**	0.00	**0.09**	0.00	0.00	0.00	0.00	**0.03**	0.00	0.00	0.00
	8-O-4'-FA	0.00	0.00	0.00	0.00	0.00	0.00	**0.23**	0.00	0.00	0.00	0.00	0.00	0.00	0.00	0.00
	std. dev.$_{\text{8-O-4'-FA}}$	0.00	0.00	0.00	0.00	0.00	0.00	**0.01**	0.00	0.00	0.00	0.00	0.00	0.00	0.00	0.00
	Tri-FA 1	0.00	0.00	0.00	0.00	0.00	0.00	0.00	0.00	0.00	0.00	0.00	0.00	0.00	0.00	0.00
	std. dev.$_{\text{TriFA 1}}$	0.00	0.00	0.00	0.00	0.00	0.00	0.00	0.00	0.00	0.00	0.00	0.00	0.00	0.00	0.00
	Tri-FA 2	0.00	0.00	0.00	0.00	0.00	0.00	**0.10**	0.00	0.00	0.00	0.00	0.00	0.00	0.00	0.00
	std. dev.$_{\text{TriFA 2}}$	0.00	0.00	0.00	0.00	0.00	0.00	**0.01**	0.00	0.00	0.00	0.00	0.00	0.00	0.00	0.00
	8-8’-furan-FA	**0.73**	**0.25**	0.00	**21.50**	**39.83**	**28.19**	**60.58**	**40.42**	**0.27**	0.00	**9.72**	**21.88**	0.00	0.00	**0.29**
	std. dev.$_{\text{8-8’-furan-FA}}$	**0.04**	**0.01**	0.00	**0.70**	**0.31**	**0.11**	**2.05**	**1.11**	**0.02**	0.00	**0.34**	**0.76**	0.00	0.00	**0.00**
	8-5'-FA	0.00	0.00	0.00	0.00	**27.81**	0.00	**78.47**	**48.48**	0.00	0.00	0.00	**76.06**	0.00	0.00	0.00
CS	std. dev.$_{\text{8-5'-FA}}$	0.00	0.00	0.00	0.00	**1.96**	0.00	**0.27**	**0.27**	0.00	0.00	0.00	**0.60**	0.00	0.00	0.00
	m/z = 401$_{\text{9.75 min}}$	0.00	**0.89**	**1.66**	**5.77**	**19.75**	**9.00**	**47.16**	**14.61**	0.00	**0.14**	**2.89**	**21.36**	**0.94**	**0.15**	**1.67**
	std. dev.$_{\text{m/z 401}}$	0.00	**0.01**	**0.07**	**0.57**	**0.11**	**0.33**	**1.64**	**0.55**	0.00	**0.01**	**0.07**	**0.50**	**0.03**	**0.01**	**0.04**
	5-5'-FA	0.00	0.00	0.00	**16.08**	**41.51**	**23.02**	**74.12**	**33.67**	0.00	0.00	0.00	**56.43**	0.00	0.00	0.00

	std. dev.$_{5-5'-FA}$	0.00	0.00	0.00	**0.20**	**0.75**	**1.45**	**3.26**	**0.11**	0.00	0.00	0.00	**1.93**	0.00	0.00	0.00
	8-O-4'-FA	0.00	0.00	0.00	**6.65**	**7.43**	**5.73**	**10.89**	**8.22**	0.00	0.00	**3.80**	**2.16**	0.00	0.00	0.00
	std. dev.$_{8-O-4'-FA}$	0.00	0.00	0.00	**0.53**	**0.03**	**0.20**	**0.50**	**0.15**	0.00	0.00	**0.28**	**0.07**	0.00	0.00	0.00
WS	8-8’-furan-FA	0.00	0.00	0.00	**3.64**	**36.45**	**28.80**	**50.71**	**36.19**	0.00	0.00	**10.59**	**9.71**	0.00	0.05	0.00
	std. dev.$_{8-8’-furan-FA}$	0.00	0.00	0.00	**1.11**	**0.77**	**0.06**	**0.92**	**0.41**	0.00	0.00	**0.12**	**1.62**	0.00	0.00	0.00
	8-5'-FA	0.00	0.00	0.00	0.00	0.00	0.00	**8.41**	0.00	0.00	0.00	0.00	0.00	0.00	0.00	0.00
	std. dev.$_{8-5'-FA}$	0.00	0.00	0.00	0.00	0.00	0.00	**0.81**	0.00	0.00	0.00	0.00	0.00	0.00	0.00	0.00
	m/z = 401$_{9.75\ min}$	0.00	0.00	0.00	**19.95**	**48.23**	**17.10**	**92.35**	**31.31**	0.00	0.00	0.00	**20.65**	0.00	0.00	0.00
	std. dev.$_{m/z\ 401}$	0.00	0.00	0.00	**0.34**	**3.04**	**0.71**	**0.10**	**1.20**	0.00	0.00	0.00	**1.32**	0.00	0.00	0.00
	5-5'-FA	0.00	0.00	0.00	**30.51**	**40.88**	**27.78**	**64.02**	**37.89**	0.00	0.00	0.00	**37.70**	0.00	0.00	0.00
	std. dev.$_{5-5'-FA}$	0.00	0.00	0.00	**0.00**	**2.52**	**0.38**	**0.54**	**0.58**	0.00	0.00	0.00	**1.81**	0.00	0.00	0.00
	8-O-4'-FA	0.00	0.00	0.00	0.00	**7.65**	0.00	**10.03**	**11.19**	0.00	0.00	0.00	0.00	0.00	0.00	0.00
	std. dev.$_{8-O-4'-FA}$	0.00	0.00	0.00	0.00	**0.59**	0.00	**0.90**	**0.16**	0.00	0.00	0.00	0.00	0.00	0.00	0.00
CSlignin	8-8’-furan-FA	0.00	0.00	0.00	**0.86**	**1.75**	**1.09**	**2.75**	**3.05**	0.00	0.00	**0.42**	**1.21**	0.00	**0.14**	0.00
	std. dev.$_{8-8’-furan-FA}$	0.00	0.00	0.00	**0.06**	**0.12**	**0.06**	**0.13**	**0.12**	0.00	0.00	**0.01**	**0.03**	0.00	**0.01**	0.00
	8-5'-FA	0.00	0.00	0.00	0.00	0.00	0.00	0.00	0.00	0.00	0.00	0.00	0.00	0.00	0.00	0.00
	std. dev.$_{8-5'-FA}$	0.00	0.00	0.00	0.00	0.00	0.00	0.00	0.00	0.00	0.00	0.00	0.00	0.00	0.00	0.00
	m/z = 401$_{9.75\ min}$	0.00	0.00	0.00	0.00	**3.51**	**0.00**	**5.97**	**4.12**	0.00	0.00	0.00	**5.01**	0.00	0.00	0.00
	std. dev.$_{m/z\ 401}$	0.00	0.00	0.00	0.00	**0.17**	**0.00**	**0.36**	**0.02**	0.00	0.00	0.00	**0.21**	0.00	0.00	0.00
	5-5'-FA	0.00	0.00	0.00	**36.59**	**61.35**	**45.24**	**74.18**	**65.32**	0.00	0.00	0.00	**97.03**	0.00	0.00	0.00
	std. dev.$_{5-5'-FA}$	0.00	0.00	0.00	**3.41**	**1.58**	**0.95**	**7.52**	**0.19**	0.00	0.00	0.00	**3.45**	0.00	0.00	0.00

Supplementary File 9: Table S6. Determined absolute values for the release of m/z $401_{10.66\ min}$ [µg/mg sample]

Enzymes	Broth	AnFaeB 1	AsFaeF 1	AnFaeC 5	AnidFAEC 5	AsFaeC 5	C1FaeA1 5	C1FaeA2 5	FoFae2 6	AsFeE 6	C1FaeB2 6	AnFaeA 7	AnFaeJ 9	AsFaeI 13	ShFae1 13
CFoligo	0.00	**0.37**	0.00	**1.48**	**5.92**	**3.57**	**13.33**	**11.14**	**1.98**	0.00	**1.36**	**6.47**	**0.25**	0.00	0.00
Std. $dev._{CFoligo}$	0.00	**0.00**	0.00	**0.03**	**0.03**	**0.23**	**0.03**	**0.78**	**0.00**	0.00	**0.06**	**0.90**	**0.01**	0.00	0.00
CS	0.00	**0.22**	0.00	**1.67**	**3.15**	**2.59**	**4.18**	**3.53**	**0.19**	**0.16**	**1.97**	**0.96**	0.00	**0.17**	0.00
Std. $Dev._{CS}$	0.00	**0.02**	0.00	**0.11**	**0.07**	**0.06**	**0.22**	**0.19**	**0.01**	**0.01**	**0.16**	**0.03**	0.00	**0.00**	0.00
WS	0.00	**0.05**	0.00	**2.24**	**2.48**	**2.12**	**3.23**	**2.35**	**0.04**	0.00	**1.56**	**1.97**	**0.05**	**0.10**	0.00
Std. $Dev._{WS}$	0.00	**0.01**	0.00	**0.04**	**0.08**	**0.03**	**0.13**	**0.06**	**0.00**	0.00	**0.02**	**0.09**	**0.00**	**0.00**	0.00
CSlignin	0.00	**0.17**	0.00	**1.19**	**2.20**	**1.94**	**1.98**	**2.49**	**0.25**	**0.18**	**1.41**	**0.35**	0.00	0.00	0.00
Std. $Dev._{CSlignin}$	0.00	**0.01**	0.00	**0.06**	**0.17**	**0.07**	**0.16**	**0.19**	**0.00**	**0.00**	**0.01**	**0.02**	0.00	0.00	0.00

References

Appeldoorn, M.M., de Waard, P., Kabel, M.A., , Gruppen, H., and Schols. H.A.. (2013). “Enzyme resistant feruloylated xylooligomer analogues from thermochemically treated corn fiber contain large side chains, ethyl glycosides and novel sites of acetylation.” *Carbohydr. Res.* 381: 33–42. doi.org/10.1016/j.carres.2013.08.024.

Crepin, V.F., Faulds C. B., and Connerton, I.F. (2004). “Functional classification of the microbial feruloyl esterases.” *Appl. Biochem. Biotechnol.* 63: 647–52. doi.org/10.1007/s00253-003-1476-3.

Dilokpimol, A., Mäkelä, M.R., M.V. Aguilar-Pontes, Benoit-Gelber, I., Hildén K.S., and de Vries, R.P. (2016). “Diversity of fungal feruloyl esterases: Updated phylogenetic classification, properties, and industrial applications.” *Biotechnol. Biofuels* 9: 231–40. doi.org/10.1186/s13068-016-0651-6.

Dilokpimol, A., Mäkelä, M.R., Mansouri, S., Belova, O., Waterstraat, M., Bunzel, M., de Vries, R.P., and Hildén, K.S. (2017). “Expanding the feruloyl esterase gene family of *Aspergillus niger* by characterization of a feruloyl esterase, FaeC.” *N. Biotechnol.* 37: 200–209. doi.org/10.1016/j.nbt.2017.02.007.

Dilokpimol, A., Mäkelä, M.R., Varriale, S., Zhou, M., Cerullo, G., Gidijala, L., Hinkka, H., et al. (2018). “Fungal feruloyl esterases: functional validation of genome mining based enzyme discovery including uncharacterized subfamilies.” *N. Biotechnol.* 41: 9–14. doi.org/10.1016/j.nbt.2017.11.004.

Englyst, H. N., and Cummings, J.H. (1984). “Simplified method for the measurement of total non-starch polysaccharides by gas-liquid chromatography of constituent sugars as alditol acetates.” *Analyst* 109: 937–42. doi.org/10.1039/AN9840900937.

Erven, G. van, Nayan, N., Sonnenberg, A.S.M., Hendriks, W.H., Cone, J.W., and Kabel, M.A. (2018). “Mechanistic insight in the selective delignification of wheat straw by three white-rot fungal species through quantitative 13C-IS Py-GC–MS and whole cell wall HSQC NMR.” *Biotechnol. Biofuels* 11: 262–78. doi.org/10.1186/s13068-018-1259-9.

Erven, G. van, de Visser, R., Merkx, D.W.H., Strolenberg, W., de Gijsel, P., Gruppen, H., and Kabel, M.A. (2017). “Quantification of lignin and its structural features in plant biomass using 13C lignin as internal standard for pyrolysis-GC-SIM-MS.” *Anal. Chem.* 89: 10907–16. doi.org/10.1021/acs.analchem.7b02632.

Faulds, C.B, and Williamson, G. (1994). “Purification and characterization of a ferulic acid esterase (FAE-III) from *Aspergillus niger*: Specificity for the phenolic moiety and binding to microcrystalline cellulose.” *Microbiology* 140: 779–87. doi.org/10.1099/00221287-140-4-779.

Thibault, J.F. (1979). “Automatisation du dosage des substances pectiques par la methode au meta-hydroxydiphenyl.” *Lebensm. Wiss. Technol.* 12: 247–51.

Hoon, K., Padmakshan, D., Li, Y., Rencoret, J., Hatfield R.D., and Ralph, J. (2017). “Characterization and elimination of undesirable protein residues in plant cell wall materials for enhancing lignin analysis by solution-state nuclear magnetic resonance spectroscopy.” *Biomacromolecules* 18: 4184–95. doi.org/10.1021/acs.biomac.7b01223.

Hoon, K., Ralph, J., and Akiyama, T. (2008). “Solution-state 2D NMR of ball-milled plant cell wall gels in DMSO-D6.” *Bioenerg. Res* 1: 56–66. doi.org/10.1039/b916070a.

Kühnel, S., Pouvreau, L., Appeldoorn, M.M., Hinz, S.W.A., Schols H.A., and Gruppen, H. (2012). “The ferulic acid esterases of *Chrysosporium lucknowense* C1: purification, characterization and their potential application in biorefinery.” *Enzyme Microb. Technol.* 50: 77–85. doi.org/10.1016/j.enzmictec.2011.09.008.

Lu, F., and J. Ralph. 2005. “Novel $\beta-\beta$-structures in lignins incorporating acylated monolignols.” *Appita Journal*, 233–37.

Mansfield, S.D., Kim, H., Lu, F., and Ralph, J. (2012). “Whole plant cell wall characterization using solution-state 2D NMR.” *Nat. Protoc.* 7: 1579–89. doi.org/10.1038/nprot.2012.064.

Ralph, S.A., Ralph, J., and Landucci, L. (2009). “NMR database of lignin and cell wall model compounds.” 2009. www.glbrc.org/databases_and_software/nmrdatabase/.

Río, J.C. del, Lino, A.G., Colodette, J.L., Lima, C.F., Gutiérrez, A., Martínez Á.T., Lu, F., Ralph, J., and Rencoret, J. (2015). “Differences in the chemical structure of the lignins from sugarcane bagasse and straw.” *Biomass and Bioenergy* 81: 322–38. doi.org/10.1016/j.biombioe.2015.07.006.

Río, J.C. del, Rencoret, J., Prinsen, P., Martínez Á.T., Ralph J., and Gutiérrez, A. (2012). “Structural characterization of wheat straw lignin as revealed by analytical pyrolysis, 2D-NMR, and reductive cleavage methods.” *J. Agric. Food Chem.* 60: 5922–35. doi.org/10.1021/jf301002n.
